# Pharmacological targeting of the novel β-catenin chromatin-associated kinase p38α in colorectal cancer stem cell tumorspheres and organoids

**DOI:** 10.1038/s41419-021-03572-4

**Published:** 2021-03-25

**Authors:** Martina Lepore Signorile, Valentina Grossi, Simone Di Franco, Giovanna Forte, Vittoria Disciglio, Candida Fasano, Paola Sanese, Katia De Marco, Francesco Claudio Susca, Laura Rosa Mangiapane, Annalisa Nicotra, Gabriella Di Carlo, Francesco Dituri, Gianluigi Giannelli, Giuseppe Ingravallo, Gianluca Canettieri, Giorgio Stassi, Cristiano Simone

**Affiliations:** 1 Medical Genetics, National Institute for Gastroenterology, IRCCS ‘S. de Bellis’ Research Hospital, 70013 Castellana Grotte (Ba), Italy; 2 Cellular & Molecular Pathophysiology Laboratory, Department of Surgical & Oncological Sciences, University of Palermo, 90127 Palermo, Italy; 3 Medical Genetics, Department of Biomedical Sciences and Human Oncology (DIMO), University of Bari Aldo Moro, 70124 Bari, Italy; 4 Department of Emergency and Organ Transplantation, Operating Unit of Pathological Anatomy, University of Bari Aldo Moro, 70124 Bari, Italy; 5 Personalized Medicine, National Institute for Gastroenterology, IRCCS ‘S. de Bellis’ Research Hospital, 70013 Castellana Grotte (Ba), Italy; 6 Department of Molecular Medicine, Sapienza University of Rome, 00161 Rome, Italy

**Keywords:** Cancer stem cells, Colorectal cancer, Post-translational modifications

## Abstract

The prognosis of locally advanced colorectal cancer (CRC) is currently unsatisfactory. This is mainly due to drug resistance, recurrence, and subsequent metastatic dissemination, which are sustained by the cancer stem cell (CSC) population. The main driver of the CSC gene expression program is Wnt signaling, and previous reports indicate that Wnt3a can activate p38 MAPK. Besides, p38 was shown to feed into the canonical Wnt/β-catenin pathway. Here we show that patient-derived locally advanced CRC stem cells (CRC-SCs) are characterized by increased expression of p38α and are “addicted” to its kinase activity. Of note, we found that stage III CRC patients with high p38α levels display reduced disease-free and progression-free survival. Extensive molecular analysis in patient-derived CRC-SC tumorspheres and APC^Min/+^ mice intestinal organoids revealed that p38α acts as a β-catenin chromatin-associated kinase required for the regulation of a signaling platform involved in tumor proliferation, metastatic dissemination, and chemoresistance in these CRC model systems. In particular, the p38α kinase inhibitor ralimetinib, which has already entered clinical trials, promoted sensitization of patient-derived CRC-SCs to chemotherapeutic agents commonly used for CRC treatment and showed a synthetic lethality effect when used in combination with the MEK1 inhibitor trametinib. Taken together, these results suggest that p38α may be targeted in CSCs to devise new personalized CRC treatment strategies.

## Introduction

Colorectal cancer (CRC) is the third most frequent malignancy but the second leading cause of death for tumor worldwide^1^. The survival rate of affected patients largely depends on the stage at which the tumor is diagnosed. About one-third of CRC patients have stage III disease, which is characterized by spread to regional lymph nodes and absence of distant metastases^2^. Stage III CRC patients are at high risk for tumor recurrence, and their overall prognosis, for which the N stage has been found to be a reliable indicator, remains unsatisfactory even with curative surgery and adjuvant chemotherapy^3^. Indeed, it is reported that more than one-third of stage III CRC patients will develop recurrence or metastasis within 5 years of systemic therapy^4^.

CRC stem cells (CRC-SCs) are involved in drug resistance, tumor recurrence, and metastasis after primary treatment. Indeed, conventional therapies wipe out bulk tumor populations, while CRC-SCs are resistant to chemotherapy and radiotherapy^5^. Therefore, new treatment approaches targeting CRC-SCs are needed in order to achieve complete tumor eradication^6^.

Several dysregulated signaling pathways confer to cancer stem cells (CSCs) a survival advantage over current therapies; among these pathways, the main driver controlling CSC fate is Wnt signaling^7^. During carcinogenesis, increasing amounts of β-catenin resulting from APC inactivation translocate into the nucleus and modulate the transcriptional activity of TCF/LEF transcription factors^8^. High levels of nuclear β-catenin lead to constitutive activation of the Wnt pathway, loss of normal cellular architecture, and neoplastic conversion^9^.

Previous reports indicate that p38α, one of the four p38 isoforms (α, β, γ, δ), is highly expressed in colorectal neoplasms compared to normal mucosa^10^ and is the main p38 isoform in colorectal and ovarian cancer cells^11–13^. Importantly, Wnts can activate p38 MAPKs. Indeed, Wnt3a was recently shown to stimulate p38 activation in mouse F9 teratocarcinoma cells. Of note, Wnt-induced p38 activation appears to regulate canonical Wnt/β—catenin signaling^8^, and p38 was found to phosphorylate GSK3β at Thr390, which inactivates GSK3β kinase activity, leading to β-catenin accumulation^14^.

Our previous results indicate that p38α is required for CRC cell proliferation and survival, and its inhibition induces growth arrest, autophagy, and cell death both in vitro and in vivo^11,15–17^. Recently, we demonstrated the existence of a p38α-ERK synthetic lethality crosstalk that is crucial for CRC therapy response. Indeed, combined inhibition of p38α and MEK1 efficiently reduced the volume of xenografted tumors and colitis-associated orthotopic tumors in vivo^10,12^. Besides, resistance to cisplatin (CDDP), irinotecan (CPT-11), and 5-fluorouracil (5-FU) chemotherapy has been shown to involve MAPK signaling, and recent studies identified p38α MAPK as a mediator of resistance to various agents in CRC patients^13^. Our previous studies also revealed that p38α inhibition sensitizes chemoresistant CRC cells to CDDP, with the combined treatment inducing Bax-dependent apoptosis in both chemosensitive and chemoresistant cells^18^.

p38α is considered a prototypical chromatin-associated kinase. Indeed, it can associate with and phosphorylate several transcription factors and can recruit subunits of the SWI/SNF ATP-dependent remodeling complexes directly to the DNA, thereby modulating chromatin structure and transcription^19^. p38α also phosphorylates MSK1, which in turn phosphorylates Ser10 on histone H3, inducing a transcriptional activation-permissive chromatin modification^20^. Additionally, p38α can physically interact with RNA polymerase II and promote the transcription elongation step^21^.

In recent years, a role has emerged for the p38 pathway in CSC regulation. Indeed, p38 seems to promote survival in hypoxic and serum-starved CRC-SCs^22^ and mediates CSC drug resistance to oxaliplatin and anti-angiogenic agents^23^. Moreover, p38-inhibited cells showed decreased expression of CSC markers and reduced sphere-forming ability in head and neck squamous cell carcinoma^24^.

Here, we performed an extensive characterization of p38α in patient-derived stage III CRC-SCs, identifying it as a direct interactor of β-catenin, the key element of the Wnt pathway. p38α acts as a chromatin-associated kinase involved in the activation of β-catenin target gene transcription, and its pharmacological manipulation affects various cancer features. Importantly, we show that targeting p38α may overcome chemoresistance in a CRC-SC model, with p38α levels being a potential new marker of therapeutic efficacy in stage III CRC patients.

## Results

### p38α is a potential marker of therapeutic efficacy in stage III CRC patients

**Fig. 1:**
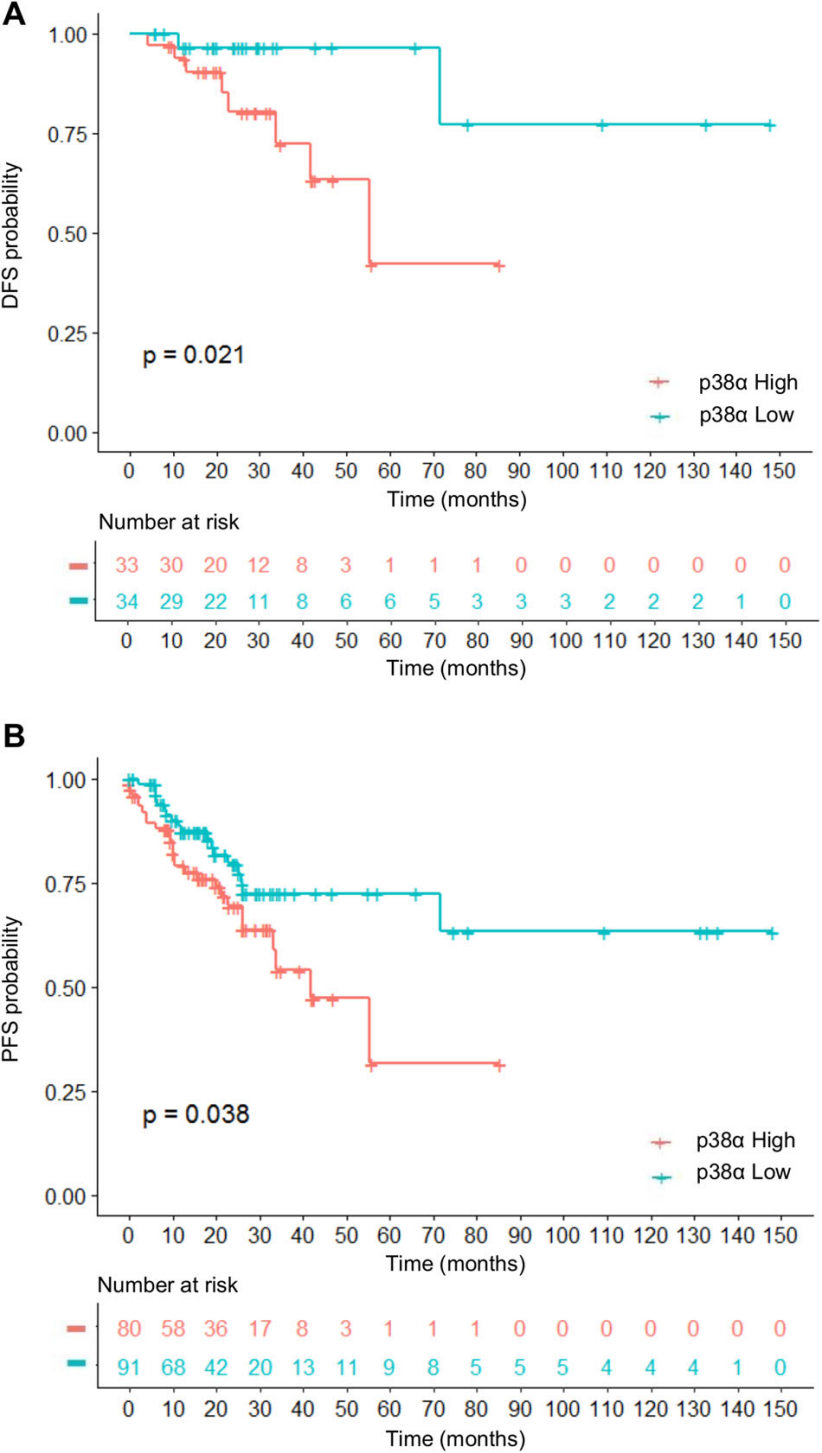
p38α is a potential marker of therapy efficacy in stage III CRC patients. **A**, **B** Kaplan–Meier curve of disease-free survival (DFS) (**A**) and progression-free survival (PFS) (**B**) in stage III CRC patients as a function of p38α levels.

Stage III CRC patients are eligible for adjuvant and combination therapies but still have a poor prognosis. In an attempt to identify potential targets for stage III disease therapy, we performed a meta-analysis on a cohort of colorectal tumor tissues retrieved from The Cancer Genome Atlas (TCGA) PanCancer Atlas. This dataset encompasses clinical data of 580 CRC patients with stage I–IV disease. We stratified the 171 stage III CRC patients based on p38α mRNA level *Z*-score and classified them as p38α high (80 patients) or p38α low (91 patients) to investigate the association between p38α expression and prognosis. We found that high expression of p38α was associated with worse disease-free survival (DFS, *p*-value = 0.0209) and progression-free survival (PFS, *p*-value = 0.0382) (Fig. 1A, B).

### Establishment and characterization of patient-derived stage III CRC-SCs

**Fig. 2:**
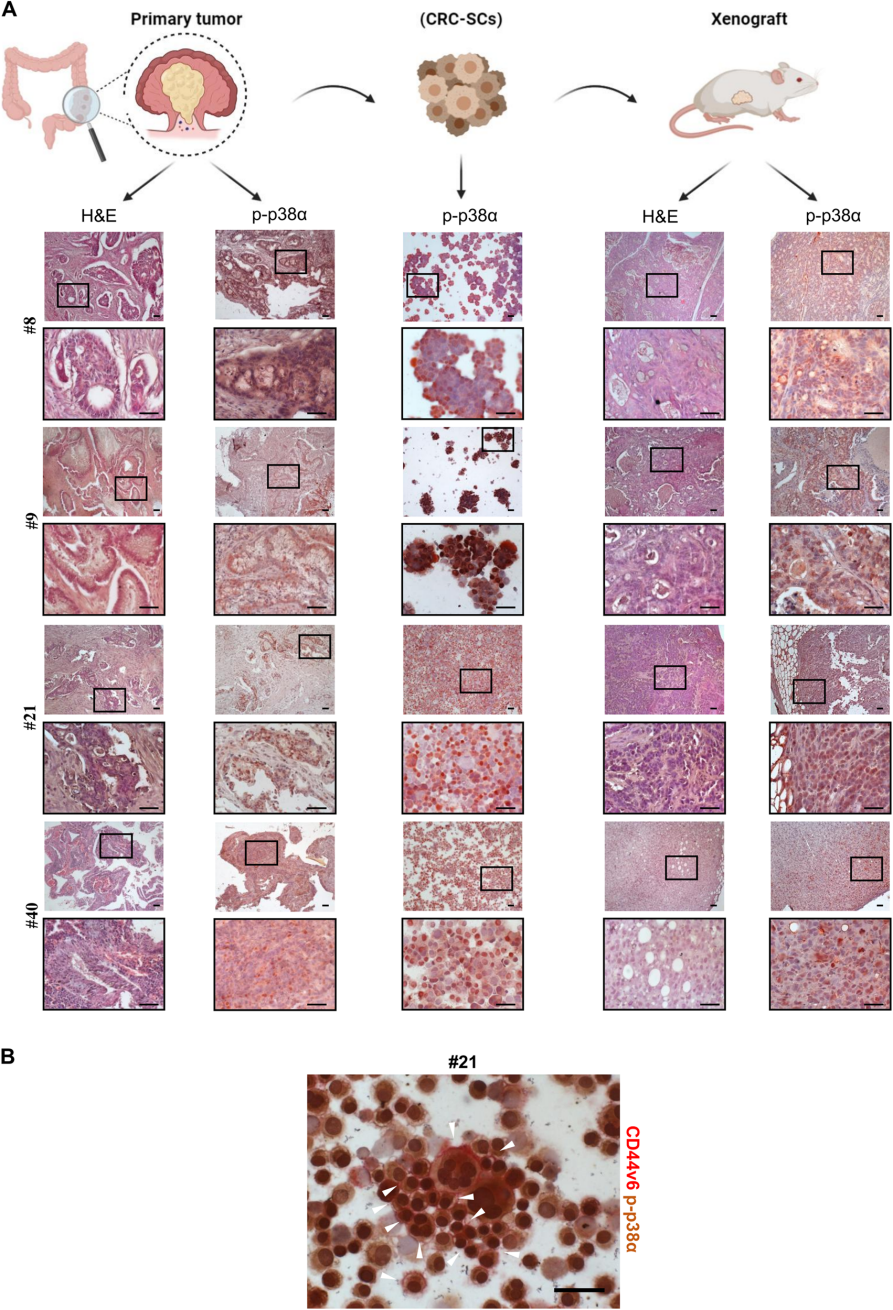
CRC-SCs recapitulate parental tumor features. **A** Representative hematoxylin and eosin staining and immunohistochemical analysis of p-p38α in primary CRC, isolated CRC-SCs, and CRC-SC-derived xenografts. Scale bars, 100 µm. **B** Representative immunocytochemical analysis of CD44v6 (red) and p-p38α (brown) in CRC-SCs #21. White arrow-heads indicate CD44v6^+^/p-p38α^+^ CRC-SCs. Scale bar, 100 µm.

Patient-derived CRC-SCs are currently used as a model to evaluate drug response^25^. We thus characterized various CRC cell lines established from stage III CRC patients and grown as tumorspheres by analyzing their mutation status, chromosomal and microsatellite instability (MSI), and expression of a group of surface markers (Fig. S1A, B). Immunoblot analysis showed increased amounts of c-Myc in patient-derived CRC cells compared to HCEC-1CT normal colonocytes and the CRC cell lines HCT116 and HT29. Notably, patient-derived CRC-SCs also proved rich in β-catenin, phospho-p38, and the stem cell markers CD44 and CD133, while expressing low levels of keratin 20, a major cellular protein found in mature enterocytes (Fig. S1B). All patient-derived CRC-SC lines (#8, #9, #21, and #40) used in this study were also found to express high levels of phospho-p38α (p-p38α, i.e., p38α-active form) (Fig. S1C). In order to characterize our CRC-SC-based pre-clinical model, we performed immunohistochemical analyses showing that CRC-SCs recapitulate parental tumor histological features and cellular heterogeneity in terms of p-p38α expression and nuclear localization. Interestingly, p-p38α expression is maintained in CRC-SCs even after the in vivo passage, as demonstrated by the analysis of tumor xenografts generated by subcutaneous injection of CRC-SCs (Fig. 2A). Importantly, our results also showed that most cells expressing CD44v6, an alternative splicing form of CD44 playing a major role in cancer progression, cell migration, and invasion^26^, are characterized by nuclear localization of p-p38α, thus suggesting that activation of p38α is crucial for CRC-SCs (Fig. 2B).

### p38α is a new functional member of β-catenin complexes

In order to characterize the functional relationship between p38α and the Wnt/β-catenin pathway in CRC, we assessed p38α and β-catenin protein localization in our colorectal model systems.

HCEC-1CT and HT29 cells were serum-starved to retain β-catenin in the cytoplasm and then switched to a serum-containing medium with or without LiCl, a well-established agonist of the Wnt/β-catenin pathway. Immunoblot analysis confirmed that expression of β-catenin and its direct target gene c-Myc is barely detectable under serum starvation, while it increases substantially after serum stimulation. Interestingly, p38α showed the same nuclear/cytoplasmic localization of β-catenin under all treatment conditions. Specifically, both were predominantly found in the nucleus in the CRC cell line, while they were primarily located in the cytoplasm in HCEC-1CT cells (Fig. S2A). These data were corroborated by immunofluorescence staining (Fig. S2B).

Experiments performed in patient-derived stage III CRC-SC tumorspheres cultured with or without a Wnt/β-catenin pathway inhibitor (PRI-724) or activator (Wnt3a alone or in combination with LiCl) confirmed p38α–β-catenin nuclear/cytoplasmic co-localization under all treatment conditions also in these cells (Fig. S2C).

**Fig. 3:**
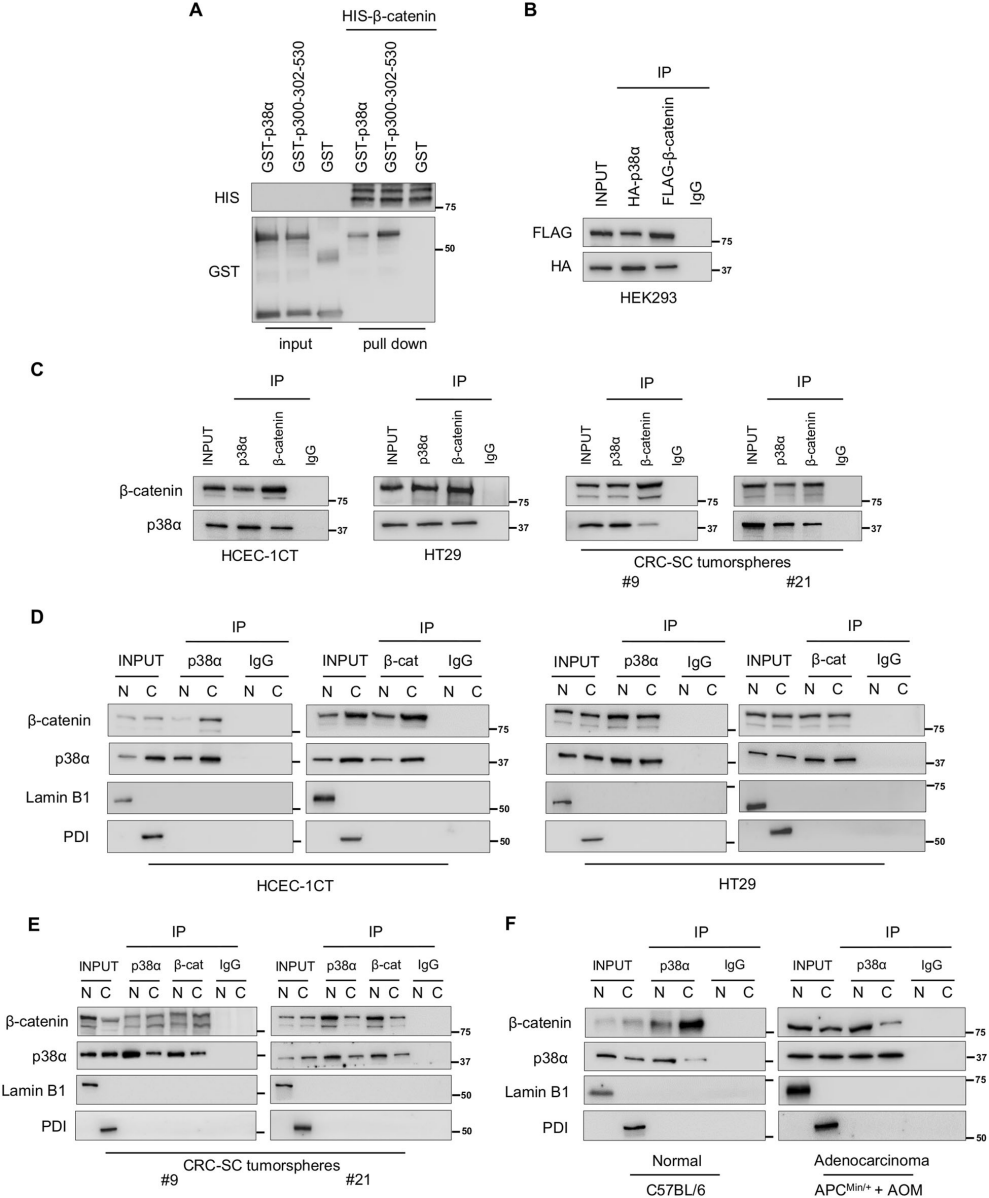
Functional interaction between p38α and β-catenin in in vitro models of CRC. **A** In vitro binding assay between GST-p38α fusion protein and HIS-β-catenin. Bound proteins were analyzed by immunoblotting using anti-GST and anti-HIS antibodies. **B** Co-immunoprecipitation with anti-HA and anti-FLAG antibodies in HEK293 cells overexpressing HA-p38α or FLAG-β-catenin. **C** Co-immunoprecipitation of endogenous p38α and β-catenin in the indicated cells. **D** and **E** Co-immunoprecipitation of endogenous p38α and β-catenin in nuclear and cytoplasmic fractions of the indicated cells. **F** Co-immunoprecipitation of endogenous p38α and β-catenin in nuclear and cytoplasmic fractions from C57BL/6 mice normal colon tissue and AOM-treated APC^Min/+^ mice adenocarcinoma tissue. Input corresponds to 10% of the lysate. Anti-IgGs were used as negative controls. Lamin B1: nuclear loading control; PDI: cytoplasmic loading control; N = nucleus, C = cytoplasm, β-cat = β-catenin.

These results prompted us to ascertain whether p38α directly interacts with β-catenin. We thus performed an in vitro-binding assay between a full-length His-tagged β-catenin recombinant protein and a GST-tagged p38α fusion protein, using GST-p300-320-530 as a positive control^27^, and found that p38α directly interacts with β-catenin in vitro (Fig. 3A).

Of note, co-immunoprecipitation (Co-IP) experiments performed in HEK293 cells transiently transfected with HA-tagged p38α and FLAG-tagged β-catenin confirmed that this interaction also occurs *in cellulo* (Fig. 3B).

Next, we evaluated whether endogenous p38α is a partner of β-catenin complexes in our colorectal model systems. Immunoprecipitation of whole-cell lysates with an antiserum against p38α or β-catenin, followed by immunoblotting, indicated that p38α is a molecular partner of β-catenin complexes in these cells (Figs. 3C; S3A).

Further immunoprecipitation experiments in HCEC-1CT, HT29 cells, and patient-derived CRC-SCs subjected to a cellular fractionation protocol revealed that p38α and β-catenin co-immunoprecipitate mainly in the cytoplasm in normal colonocytes but predominantly in the nucleus in CRC cells and patient-derived CRC-SCs (Fig. 3D, E; S3B).

To validate the results obtained *in cellulo*, we performed in vivo experiments in APC^Min/+^ mice, which are heterozygous for a missense mutation in the APC gene and model human familial adenomatous polyposis (FAP) as they develop multiple intestinal polyps that acquire carcinoma features after exposure to the carcinogen drug azoxymethane (AOM)^28^. We found increased levels of p-p38, p-ERK, β-catenin, and c-Myc in mouse colon adenoma and adenocarcinoma compared to normal colon mucosa (Fig. S3C). Moreover, p38α and β-catenin co-immunoprecipitated mainly in cytoplasmic fractions in normal colon mucosa but mostly in nuclear fractions in adenocarcinoma tissue (Figs. 3F; S3D).

### p38α is a novel β-catenin chromatin-associated kinase

**Fig. 4:**
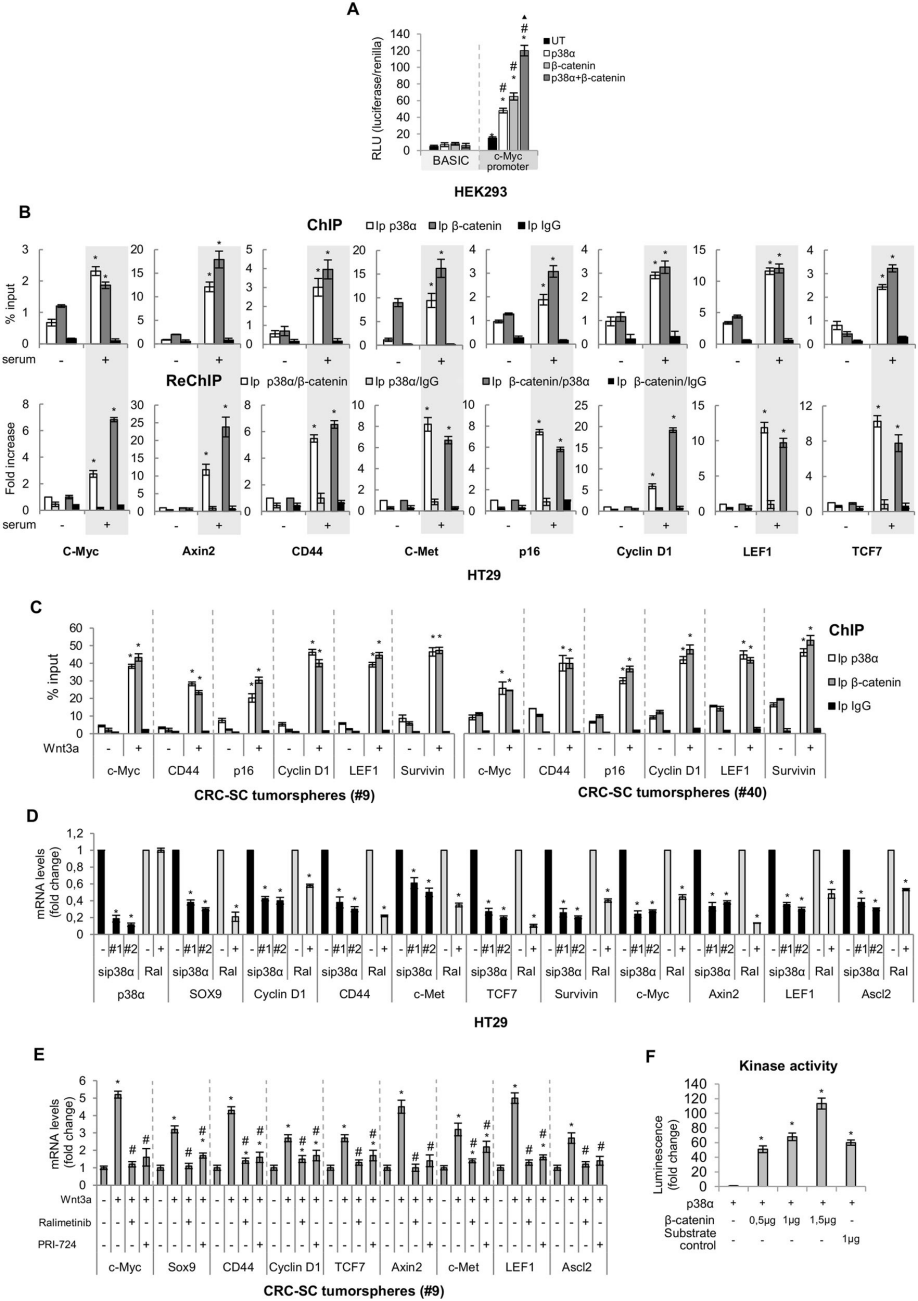
p38α is a novel β-catenin chromatin-associated kinase. **A** Luciferase assay for c-Myc promoter activity. HEK293 cells were serum-starved for 24 h and transfected with either the empty vector (pcDNA) or pcDNA3.1-HAHA-p38α and/or pcDNA-β-catenin expression constructs. **B** Chromatin immunoprecipitation (ChIP) and re-ChIP assays in HT29 cells. Cells were serum-starved for 48 h and then switched to a serum-containing medium for 4 h. In ChIP assays (upper panels), chromatin was pulled down with anti-p38α and anti-β-catenin antibodies. In re-ChIP assays (lower panels), chromatin was pulled down with anti-p38α antibodies and then re-immunoprecipitated with anti-β-catenin antibodies and vice versa. Anti-IgGs were used as negative controls. **C** ChIP with anti-p38α and anti-β-catenin antibodies. CRC-SC tumorspheres were treated or not with Wnt3a (50 ng/ml) for 4 h. **B**, **C** Quantification was done using the % input method. **D** Real-time PCR analysis of β-catenin target genes in HT29 cells treated with ralimetinib (10 µM) or two p38α-specific siRNAs (sip38α #1 and #2) for 48 h. **E** Real-time PCR analysis of β-catenin target genes in CRC-SC tumorspheres treated with Wnt3a (50 ng/ml) for 4 h with or without ralimetinib (10 µM) or PRI-724 (25 nM) for 20 h. **D**, **E** Data are presented as mRNA fold change vs. control. **F** In vitro kinase assay showing β-catenin phosphorylation by p38α. UT = untransfected. **A** *P < 0.05 vs. BASIC, ^#^*P* < 0.05 vs. untransfected cells, ^▴^*P* < 0.05 vs. p38α-transfected or β-catenin-transfected cells. **B** *P < 0.05 vs. serum-starved cells. **C**, **E** *P < 0.05 vs. untreated cells, and ^#^*P* < 0.05 vs. Wnt3a-treated cells. **D** *P < 0.05 vs. control (DMSO or control siRNA). **F** *P < 0.05 vs. active p38α.

Based on the above findings, it is reasonable to speculate that p38α may be involved in β-catenin transcriptional activity in the nucleus of cancer cells. To confirm this hypothesis, we performed a dual-luciferase reporter assay on a c-Myc promoter-Luc construct^29^. Intriguingly, overexpression of p38α in HEK293 cells significantly enhanced transcriptional activity in a manner comparable to β-catenin overexpression. Moreover, concomitant overexpression of both proteins further increased c-Myc transcriptional activity (Fig. 4A).

To investigate the functional role of p38α and β-catenin complexes in transcriptional regulation, we then evaluated p38α and β-catenin co-occupancy of various β-catenin target gene promoters by chromatin immunoprecipitation (ChIP). HT29 cells were serum-starved to inhibit β-catenin activity in the nucleus and then switched to a serum-containing medium. ChIP assays revealed that serum mitogens dramatically stimulated β-catenin and p38α recruitment to Wnt responsive elements (WREs) of several β-catenin target genes, including c-Myc, c-Met, Survivin, and CD44, which are all involved in CRC progression (Fig. 4B). Re-ChIP experiments were then performed to confirm β-catenin and p38α co-occupancy, providing evidence that these proteins bind to the same chromatin regions after serum stimulation (Fig. 4B). Similar results were obtained in CRC-SCs, which showed co-recruitment of p38α and β-catenin on β-catenin-binding motifs of all analyzed target genes. This occurred to an even higher extent when cells were cultured with the Wnt pathway activator Wnt3a. These data suggest that p38α supports β-catenin in the activation of β-catenin target gene transcription in these cells (Figs. 4C; S3E).

Subsequently, we investigated the effect of p38α pharmacological inhibition (with the selective inhibitor ralimetinib) or genetic ablation (with two specific siRNAs) on the regulation of β-catenin target gene expression. Real-time PCR experiments showed that treatment of HT29 cells with ralimetinib or specific siRNAs leads to the downregulation of β-catenin target genes, including CD44 and Cyclin D1 (cell cycle markers), Survivin (apoptosis inhibition), c-MET (migration and invasion), and SOX9 and TCF7 (CSC proliferation markers) (Fig. 4D). These data were confirmed in patient-derived CRC-SCs treated with Wnt3a and/or ralimetinib and/or the Wnt pathway inhibitor PRI-724, and suggest that p38α is involved in the activation of β-catenin target gene transcription in CRC cells and patient-derived CRC-SC tumorspheres (Fig. 4E).

β-catenin transcriptional activity is regulated by well-known phosphorylation signals in the N-terminus and C-terminus regions^30^. We thus searched for novel β-catenin residues that could be directly phosphorylated by p38α. Since 85% of the p38α phosphorylation sites described so far are Ser-Pro or Thr-Pro motifs^31^, we performed an in silico phosphorylation prediction analysis with DISPHOS 1.3, NETPHOS 3.1, Phosida, iPTMnet, and Phosphosite Plus servers, focusing on serine and threonine residues. We identified eight putative phosphosites (S47, S129, S179, S222, T472, T547, S680, and S721) that were recognized by at least four prediction servers (Fig. S4). Of note, many of these residues have been described as being phosphorylated in vivo in different human cancers^32,33^. These findings suggest that β-catenin may be a substrate of p38α. To verify this hypothesis, we performed an in vitro kinase assay using purified proteins. Our results showed that active p38α can efficiently phosphorylate β-catenin (Fig. 4F). Furthermore, we carried out Co-IP studies to ascertain whether activation of p38α is required for the physical interaction with β-catenin in CRC-SC tumorspheres. Our results showed that p38α active form (p-p38α) interacts with β-catenin and p38α pharmacological inhibition with ralimetinib does not prevent the formation of the complex (Fig. S5A–C).

### p38α inhibition downregulates CRC-SC markers in an in vivo model

**Fig. 5:**
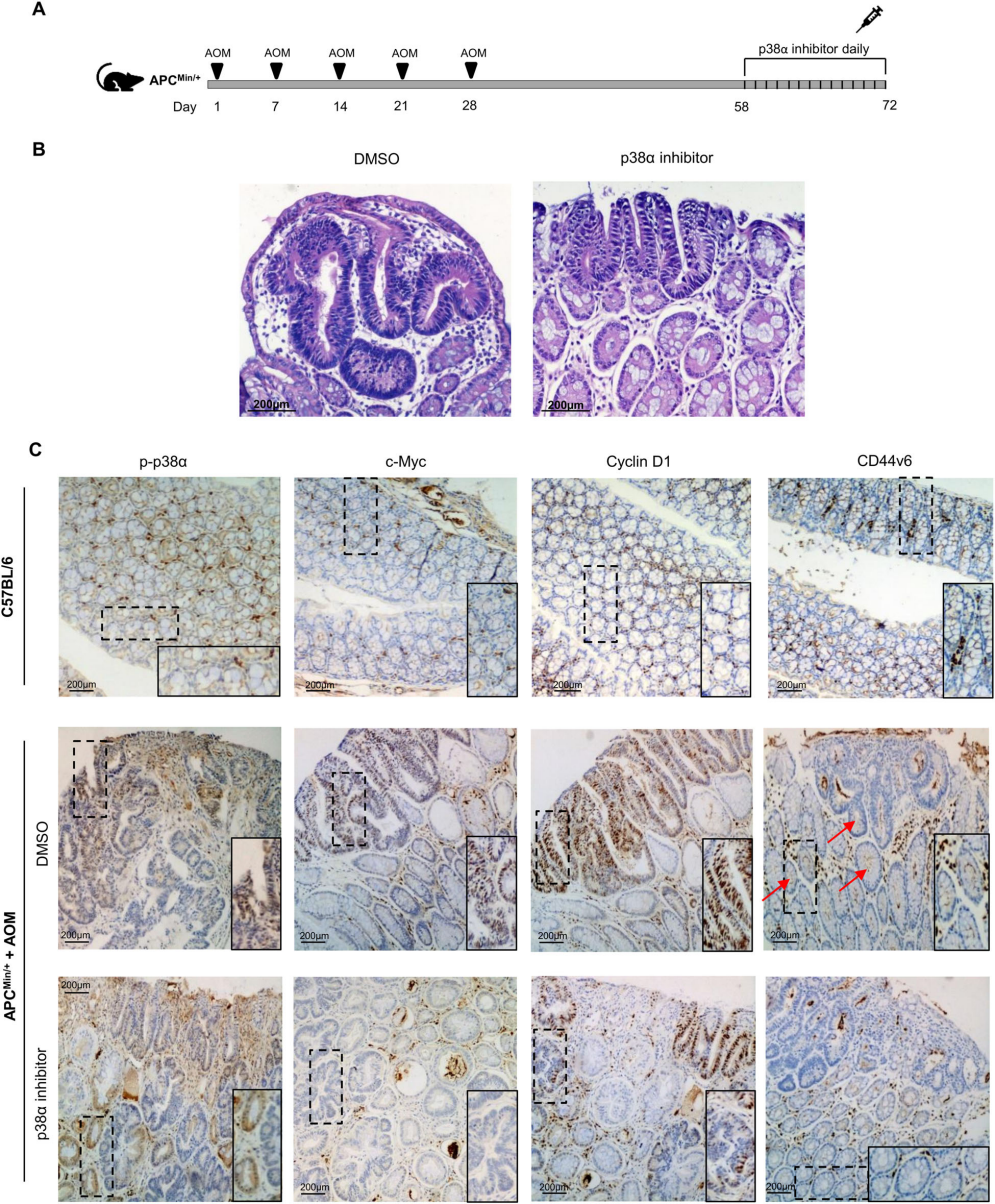
p38α inhibition downregulates CRC-SC markers in an in vivo model. **A** Mice treatment scheme. **B** Hematoxylin and eosin staining of AOM-treated APC^Min/+^ mice injected with the p38α inhibitor SB202190 or DMSO. Original magnification: 200x. **C** Immunohistochemistry analysis of colon tissue sections from C57BL/6 and AOM-treated APC^Min/+^ mice injected with the p38α inhibitor SB202190 or DMSO. Original magnification: 100x.

We previously detected a significant reduction in tumor size in the small intestine and colon of APC^Min/+^ mice treated with the p38α inhibitor SB202190^11^. Moreover, we observed malignant regression, with foci of inflammatory cells replacing adenomatous glands, in tumors of treated animals^10^. Thus, to further explore the clinical potential of p38α pharmacological inhibition for β-catenin target gene downregulation, we performed in vivo experiments in this murine model. Four-month-old animals were administered with AOM (14 mg/kg body weight) once a week for 5 weeks; one month later, they were subjected to daily intraperitoneal injections of SB202190 (0.05 μmol/kg body weight) or DMSO for 14 days and then sacrificed (Fig. 5A). Analysis of hematoxylin and eosin-stained colon sections revealed the presence of several variably pedunculated adenomatous polyps in DMSO-treated APC^Min/+^ mice, with most glands showing irregular margins and stratified pencil-shaped nuclei of various sizes. In contrast, intestinal polyps detected in SB202190-treated animals were not pedunculated, and an overall regression of adenomatous morphology was observed (Fig. 5B). Immunohistochemical analysis of healthy colon sections from C57BL/6 control mice showed no nuclear p-p38α or c-Myc staining, while cyclin D1 expression was limited to gland pits, and sporadic staining was detected for CD44v6. Conversely, in AOM-treated APC^Min/+^ mice colon sections, p-p38α staining showed high nuclear positivity in vehicle-injected animals, whereas decreased expression was detected in epithelial cells of SB202190-treated animals. Importantly, considerable neoplastic regression was observed in SB202190-injected mice colon tumors. Histopathological analysis also revealed significantly detectable neutrophilic and lymphoid infiltrates in all tumors treated with the p38α inhibitor. Moreover, nuclear c-Myc and cyclin D1 staining was detected in colon sections from vehicle-treated mice, while colon tumors from SB202190-injected animals showed faint cytoplasmic positivity with a stronger reduction in nuclear areas. In control tumor samples, staining for CD44v6 was observed at the bottom of intestinal crypts, where CRC-SCs reside, while no staining was detected in the crypts from tumors treated with SB202190 (Fig. 5C). These results confirmed that p38α pharmacological inhibition induces the downregulation of CRC-SC markers, which likely reflects a reduction in the resistant tumor cell population. The above data further strengthen the potential of p38α inhibition in CRC in vivo.

### Targeting p38α in patient-derived CRC-SCs to circumvent chemoresistance

**Fig. 6:**
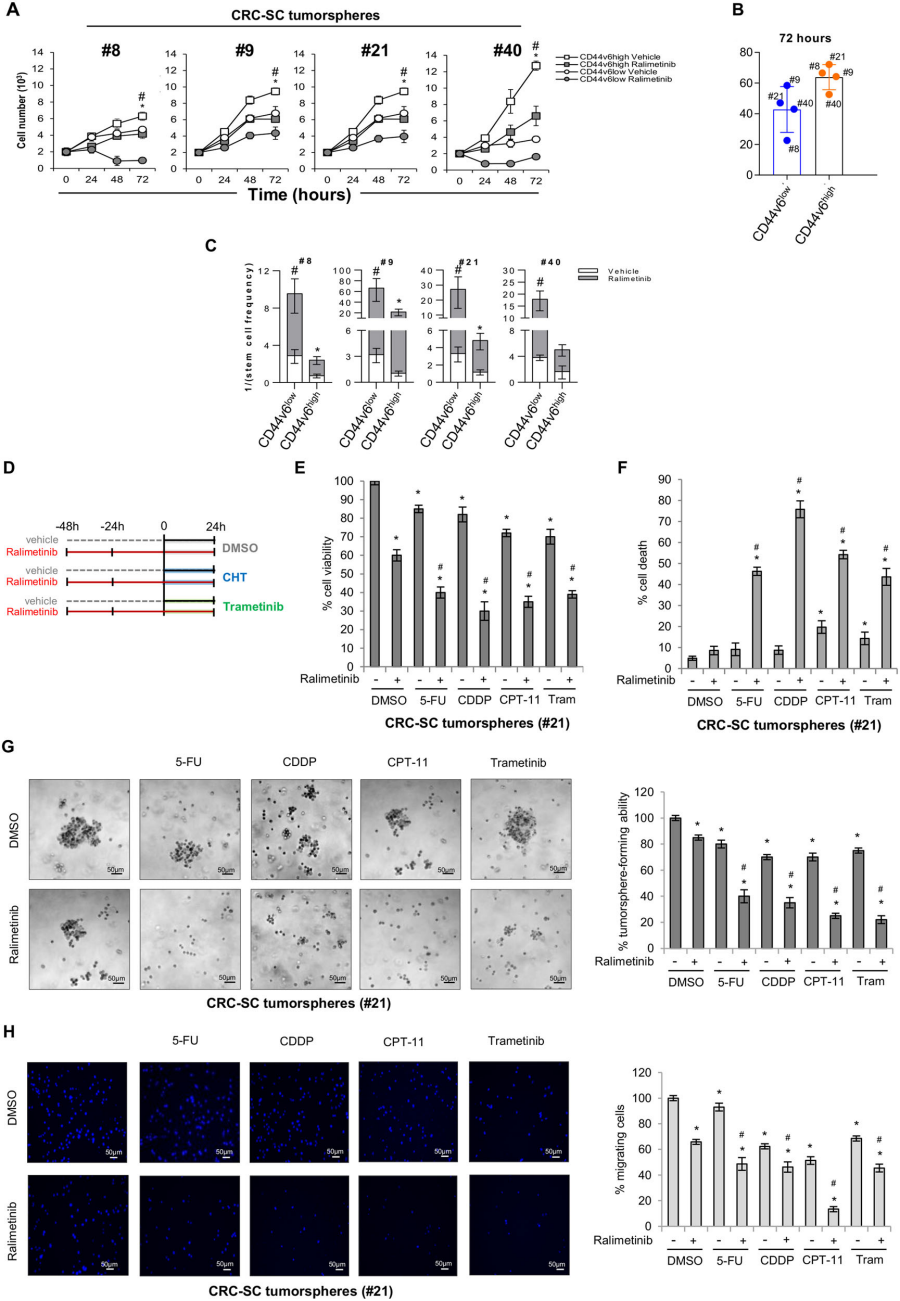
Targeting p38α in patient-derived stage III CRC-SCs to circumvent chemoresistance. **A** Growth kinetics of CD44v6^low^-enriched and CD44v6^high^-enriched CRC-SCs treated with ralimetinib (10 μM) or the vehicle for up to 72 h. **B** Viable cell number variation in CD44v6^low^- and CD44v6^high^-enriched CRC-SCs treated with ralimetinib (10 μM) for 72 h. Values were normalized against those of vehicle-treated cells. **C** Limiting dilution assay performed on CD44v6^low^-enriched and CD44v6^high^-enriched CRC-SCs. The graph shows the clonogenic capacity of each cell subset. **A**–**C** CD44v6^low^ and CD44v6^high^ represent cell samples enriched for the top 20% cells with the lowest and highest expression of CD44v6, respectively. **A**, **C** *P < 0.05: CD44v6^high^ treated with ralimetinib vs. CD44v6^high^ treated with the vehicle; and ^#^*P* < 0.05: CD44v6^low^ treated with ralimetinib vs. CD44v6^low^ treated with the vehicle. **D** Treatment scheme: CRC-SCs were pre-treated with ralimetinib (10 μM) for 48 h and then treated with 5-FU (2 μM), CDDP (30 μM), CPT-11 (30 μM), or trametinib (1 nM) for another 24 h in the presence of ralimetinib. **E** Quantification of cell viability by Cell Titer Glo in CRC-SCs #21 treated as described in (**D**). **F** Quantification of cell death by trypan blue staining in CRC-SCs #21 treated as described in (**D**). **G** Colony-forming ability of CRC-SCs #21 seeded onto double-layer soft agar and treated as described in (**D**). Data represent the percentage of colonies relative to DMSO-treated cells. Original magnification: 100x. **H** Migratory ability of growth factor-starved CRC-SCs #21 placed in the inner chamber of transwell plates and treated with the indicated compounds for 16 h. Migrating cells were fixed and counted under a fluorescence microscope. Original magnification: 100x. Tram = trametinib. *P < 0.05: treatment vs. control (DMSO); and ^#^*P* < 0.05: combined treatment vs. corresponding single treatments.

CRC-SC cultures are heterogeneous and comprise variable amounts of differentiated and CSC populations^26^. In order to evaluate the specific effect of p38α inhibition on CSCs versus differentiated cells in CRC-SC cultures, cell samples enriched for the top 20% CD44v6^high^ or CD44v6^low^ subsets by cell sorting (Fig. S6) were treated with ralimetinib and scored for viability and clonogenic potential. Our results showed that pharmacological inhibition of p38α reduces the proliferative capacity of both the CSC and the differentiated/progenitor cell compartments, identified as CD44v6^high^ and CD44v6^low^, respectively (Fig. 6A, B). Importantly, our results also indicate that pharmacological inhibition of p38α significantly reduced the clonogenic potential of both cell subsets (Fig. 6C).

Since CSCs are involved in drug resistance and disease recurrence, we evaluated the potential of ralimetinib as a sensitizing agent in chemoresistant CRC-SCs as part of a synergistic approach with currently used chemotherapeutics (CHTs), such as 5-FU, CDDP, CPT-11, or trametinib, a MEK1 inhibitor that is already approved for clinical use (Fig. 6D).

To this end, CRC-SCs pre-treated with ralimetinib for 48 h were subsequently treated with CHTs/trametinib for 24 h. Our results revealed that the combined therapeutic strategy (ralimetinib + CHTs/trametinib) has nonlinear cumulative effects and is more effective than CHTs/trametinib alone. Indeed, pre-treatment with ralimetinib reduced CRC-SC proliferative index (Figs. 6E; S7A) and increased cell death (Figs. 6F; S7B). These data support the potential of p38α inhibition to enhance sensitivity to CHTs.

Then, we performed a soft agar assay to assess the ability of patient-derived CRC-SCs to form colonies of anchorage-independent tumor cells. Our results showed that combined treatment with ralimetinib and CHTs or trametinib almost completely abolishes CRC-SC clonogenic activity compared to each single treatment (Figs. 6G; S7C).

Since patient-derived CRC-SCs grow as spheres, we also performed a spheroid-based migration assay to assess their invasive capacity. We found that co-treatment with ralimetinib and CHTs or trametinib leads to a remarkable decrease in CRC-SC migratory ability (Figs. 6H; S7D).

**Fig. 7:**
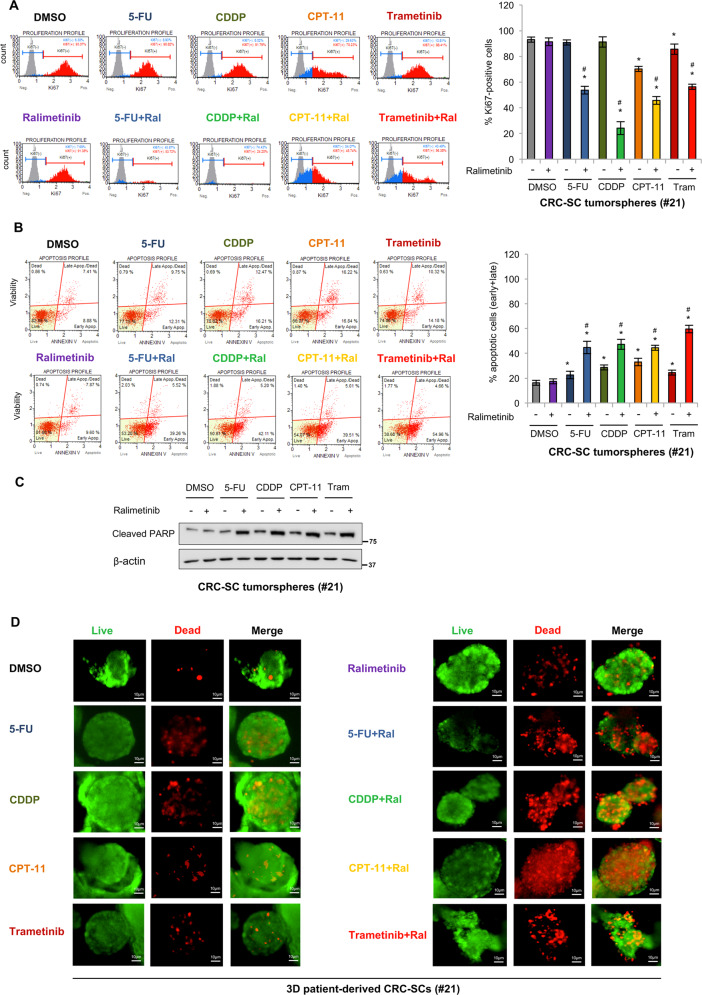
Combined treatment with ralimetinib and chemotherapeutics or trametinib has a synergistic cytotoxic effect. **A** Flow cytometry analysis of Ki67 expression in CRC-SCs #21 treated as described in Fig. 6D. Populations were gated identically using the unstained background populations shown in gray behind the Ki67-negative (blue) and Ki67-positive (red) populations. The graph on the right summarizes the percentage of Ki67-positive cells. **B** Flow cytometry analysis of annexin V staining in CRC-SCs #21 treated as described in Fig. 6D. The graph on the right summarizes the percentage of apoptotic cells (early + late). **C** Immunoblot analysis of cleaved PARP levels in CRC-SCs #21 treated as described in Fig. 6D. β-actin was used as a loading control. **D** Live/dead staining of CRC-SCs #21 grown as 3D cultures and treated as described in Fig. 6D. Tram = trametinib. Green: live cells; red: dead cells. *P < 0.05: treatment vs. control (DMSO); and ^#^*P* < 0.05: combined treatment vs. corresponding single treatments.

We further investigated the biological impact of co-treatment with ralimetinib and CHTs or trametinib on patient-derived CRC-SC fate by analyzing Ki67 expression and annexin V staining by flow cytometry. Ki67 is commonly used as a marker of cell proliferation; in addition, it is involved in the maintenance of the stem cell niche and thus can also be used as a CSC marker^34^. Based on our results, pre-treatment with ralimetinib enhanced the growth-inhibitory activity of CHTs and trametinib (Figs. 7A; S7E), and this effect was associated with induction of apoptosis, while no necrosis was observed (Figs. 7B; S7F). Activation of the apoptotic pathway in co-treated CRC-SCs was further confirmed by immunoblotting for cleaved PARP (Fig. 7C).

As additional evidence, a live/dead staining assay performed on patient-derived CRC-SC tumorspheres cultured in Matrigel showed a marked reduction in cell survival upon combined treatment with ralimetinib and CHTs or trametinib in this reconstituted 3D culture system (Fig. 7D).

Overall, these data indicate that p38α inhibition makes chemoresistant patient-derived CRC-SCs more sensitive to 5-FU, CDDP, CPT-11, and trametinib, and prone to apoptosis.

### Targeting p38α as part of a synthetic lethality approach in APC^Min/+^ mice intestinal organoids

The above preclinical data, along with previous evidence showing the existence of a p38α-ERK synthetic lethality crosstalk that is crucial for CRC therapy response^10,12^, support further investigation of a CRC-SC-targeted synthetic lethality approach based on p38α inhibition.

**Fig. 8:**
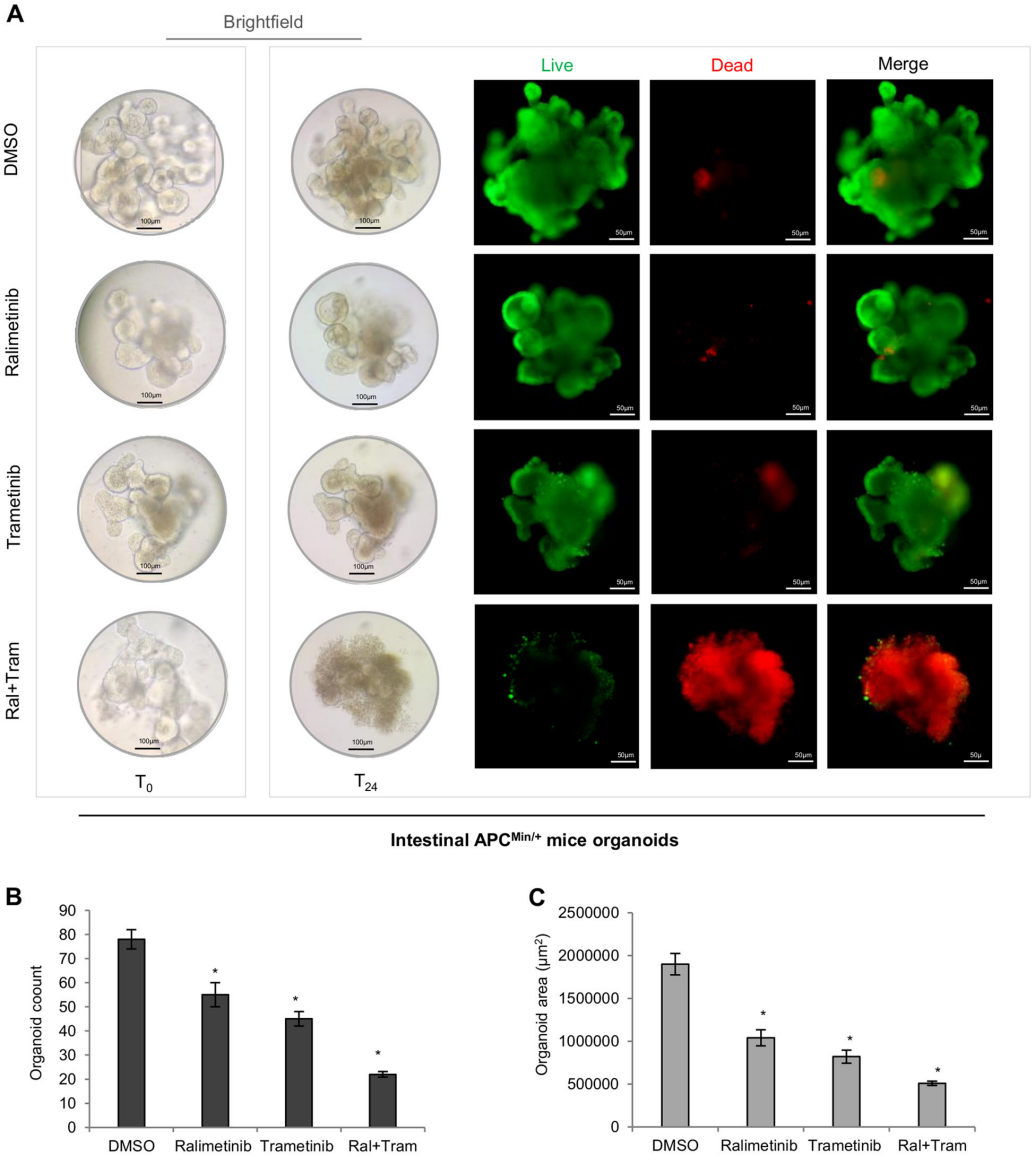
Targeting p38α as part of a synthetic lethality approach in APC^Min/+^ mice intestinal organoids. **A** Brightfield imaging of organoids formation from single adenoma intestinal crypts isolated from APC^Min/+^ mice at T0 and after 24 h treatment with ralimetinib (10 μM) and/or trametinib (1 nM). Treated organoids were also subjected to live/dead staining. Green: live cells; red: dead cells. **B** Quantification of APC^Min/+^ mice intestinal organoids after 24 h treatment with ralimetinib (10 μM) and/or trametinib (1 nM). **C** Average area of APC^Min/+^ mice intestinal organoids after 24 h treatment with ralimetinib (10 μM) and/or trametinib (1 nM), as measured using ImageJ software. *P < 0.05: treatment vs. control (DMSO).

Budding organoids formed from adenoma crypt cells of APC^Min/+^ mice were thus used to assess the survivability of intestinal stem cells after treatment with ralimetinib and trametinib. Live/dead staining revealed the synthetic lethality effect of p38α and MEK1 combined inhibition (Fig. 8A). These data were confirmed by the reduced number (Fig. 8B) and size (Fig. 8C) of organoids after treatment with both inhibitors.

## Discussion

CRC is a leading cause of cancer-related death, with a 5-year survival rate of 5% in metastatic patients^3^. Recurrence and metastasis depend on a small subset of cells within the tumor, called CSCs^5^, which are exposed to selective pressure^35^ and retain the potential for self-renewal, differentiation, and tumorigenicity^36^. Indeed, current therapies are generally based on drugs that affect rapidly dividing cells, while CRC-SCs show a low proliferative potential^37^. Furthermore, CRC-SCs display alterations of DNA repair mechanisms and express high levels of proteins that are involved in CHT resistance^38^. Hence, targeting this specific cell subpopulation may be an effective strategy to eradicate CRC and increase the survival of metastatic patients.

Several approaches focused on the CSC population are currently being evaluated, some of which successfully entered clinical trials (e.g., ClinicalTrials.gov ID NCT01190345, NCT01440127).

Here, we identified p38α as a new druggable member of β-catenin chromatin-associated kinase complexes in colorectal model systems (normal colonocytes, CRC cell lines, and patient-derived CRC-SCs that recapitulate parental tumor histological features). Moreover, we showed that CRC cells and patient-derived CRC-SCs have higher levels of activated p38α than normal colonocytes and are “addicted” to p38α activity.

Interestingly, our meta-analysis on a cohort of colorectal tumors retrieved from TCGA PanCancer Atlas dataset correlated p38α mRNA levels to stage III disease prognosis, with high p38α expression being associated with worse DFS and PFS. Based on these findings, p38α may be used as a marker of resistance and a predictor of therapy response in CRC.

We also demonstrated that p38α acts as a β-catenin chromatin-associated kinase involved in tumor proliferation, metastatic dissemination, and chemoresistance. Indeed, our data suggest that p38α serves as a regulator of gene expression by interacting with β-catenin-TCF/LEF transcriptional complexes that are recruited to WREs. The recruitment of protein kinases to promoter regulatory elements can have important functional consequences; in particular, these proteins may represent new therapeutic targets in cancer cell signaling.

Our analysis of p38α recruitment on β-catenin target gene promoters using ChIP re-ChIP assays suggests that the Wnt signaling pathway likely converges on p38α and β-catenin, which co-regulate target gene expression. Indeed, p38α feeds into the Wnt pathway at least in two different ways: one in the cytoplasm, at the level of GSK3β, as demonstrated by Thornton and colleagues^37^, and the other at the chromatin level, by interacting with and phosphorylating β-catenin on WREs, as shown by our data.

Activation of p38α and nuclear β-catenin is observed in many tumors, and several genes targeted by these signaling pathways are crucial for cancer development and progression. For instance, it has been demonstrated that c-Myc, the main Wnt/β-catenin target, is consistently overexpressed in CRC-SCs and its downregulation suppresses CRC-SC self-renewal and xenograft growth^39^. Interestingly, we found that p38α pharmacological inhibition does not prevent the formation of the p38α–β-catenin complex but downregulates several β-catenin target genes, including c-Myc, in a manner comparable to the Wnt/β-catenin inhibitor PRI-724, which is currently being evaluated in clinical trials (e.g., ClinicalTrials.gov ID NCT04351009). Moreover, our results revealed that both the CSC and the differentiated cell compartments of CRC-SC cultures are efficiently targeted by treatment with the p38α kinase inhibitor ralimetinib.

In our model systems, the combined use of CHTs and ralimetinib proved more effective than CHTs alone.

In particular, ralimetinib promoted sensitization of patient-derived CRC-SCs to CHTs commonly used in CRC therapy, such as 5-FU, CDDP, and CPT-11, as shown by a reduction in Ki67-positive cells and induction of apoptosis. These combined treatments also affected key CRC-SC features that contribute to tumor aggressiveness and metastatization, including their colony-forming and migratory ability. Moreover, ralimetinib showed a synthetic lethality effect when used in combination with the MEK1 inhibitor trametinib in patient-derived CRC-SC tumorspheres, in a human 3D culture system, and in APC^Min/+^ mice organoids.

Overall, our results confirmed the crucial role of the p38α–β-catenin axis in the regulation of intestinal tumorigenesis, suggesting that p38α manipulation could be an effective therapeutic approach in stage III CRC patients. Indeed, it may be used to target CRC-SCs in addition to bulk tumor populations to counter uncontrolled proliferation, metastatic dissemination, and chemoresistance. Several p38α inhibitors passed phase I clinical trials and are currently in phase II or III for inflammatory diseases and cancer^40–45^. In particular, ralimetinib displayed a tolerable safety profile, with preliminary evidence of antineoplastic activity in a recent phase I trial (ClinicalTrials.gov ID NCT01393990, completed in March 2020) in patients with advanced or metastatic cancer. Moreover, it is currently being tested in combination with other agents in an ongoing phase I study in CRC patients (ClinicalTrials.gov ID NCT02860780).

Altogether, our preclinical data support further clinical development of ralimetinib as a sensitizing agent to commonly used CHTs and suggest the potential of a synthetic lethality approach based on p38α and MEK1 inhibition.

## Materials and methods

### Clinical data

CRC tissues were obtained from four patients at the time of resection, in accordance with the ethical standards of the Institutional Committee on Human Experimentation (authorization CE9/2015, Policlinico P. Giaccone, Palermo) after informed consent.

### Cell lines and intestinal 3D models

HCT116 and HT29 cells were cultured in DMEM (#11360-070, Gibco) with 10% FBS (#0270-106, Gibco) and 100 IU/ml penicillin–streptomycin (#15140-122, Gibco). HEK293 cells were supplemented with 1% pyruvate (#11360070, Gibco) and 1% NEAA (#11140, Sigma-Aldrich). HCEC-1CT cells were cultured in COLO-UP (Evercyte) medium supplemented with 100 IU/ml penicillin–streptomycin (#15140-122, Gibco). #8, #9, #21, and #40 cells were isolated and propagated from CRC patients as previously described^38^. All cell lines were tested to be mycoplasma-free (#117048; Minerva Biolabs). Human intestinal 3D cultures were generated from CRC-SCs as previously described^46^. Mouse intestinal organoids were generated from APC^Min/+^ male mice as previously described^47,48^. All cell cultures were performed in a 37 °C and 5% CO_2_ incubator.

### Chemicals

5-FU (F6627), Wnt3a (H17001), LiCl (L9650), and Trypan blue (T8154) were purchased from Sigma-Aldrich. Cisplatin (S1166), Ralimetinib (S1494), Trametinib (S2673), Irinotecan (S2217), PRI-724 (S8262), and SB202190 (S1077) were purchased from SelleckChem.

### Cloning and plasmids

Efficiency DH5α Competent Cells (C2987H) and BL21 (DE3) Competent *E. coli* (C2527I) were purchased from New England Biolabs and were used for all cloning experiments. Cells were grown in standard LB media. Plasmids were generated as previously described^49^. The pcDNA-human-β-catenin (#16828) plasmid was purchased from Addgene. Primer sequences are listed in Supplementary Table 1.

### Cell transfection and RNA interference

HEK293 cells were transfected with mammalian expression plasmids using Lipofectamine 3000 (#L3000015, Thermo Fisher Scientific) according to the manufacturer’s instruction. For RNA interference, HT29 cells were transfected with 50 nM validated siRNAs (Ambion) directed against MAPK14 using the HiPerfect reagent (#301704, QIAGEN) according to the manufacturer’s instructions. siCTRL (Eurofins) was used as a non-silencing control. siRNA sequences are listed in Supplementary Table 1.

### Recombinant protein expression/purification

BL21 competent cells, transformed with different constructs, were grown in LB medium with antibiotics and induced with IPTG. Cells were lysed with B-PER lysis buffer (#78248, Thermo Fisher Scientific). GST-fusion proteins were purified with Pierce Glutathione Magnetic Agarose Beads (TH269836, Thermo Fisher Scientific) according to the manufacturer’s instructions. His-fusion proteins were purified with Dynabeads His-Tag Isolation and Pulldown (10104D, Thermo Fisher Scientific) according to the manufacturer’s instructions.

### Immunoblotting

Nuclear and cytoplasmic fractions were obtained by using the Nuclear Extraction Kit (#ab113474, Abcam) according to the manufacturer’s instructions. Immunoblots were carried out as previously described^10^. Primary antibodies: anti-β-actin (#3700), anti-c-Myc (#9402), anti-p44/42 MAPK (Erk1/2) (#9102), anti-phospho p44/42 MAPK (Erk1/2) (Thr202/tyr204) (#9106S), anti-p38 MAPK (#9212), anti-p38α MAPK (#9228), anti-phospho-p38 MAPK (Thr180/Tyr182) (#9211), anti-Lamin B1 (#12586), anti-Keratin20 (BK13063S), anti-PDI (#2446S), anti-β-catenin (#9562), anti-GST (#2625), anti-CD133 (#5860), anti-CD44 (#3570), anti-Musashi (#2154) all from Cell Signaling Technologies, anti-HIS-tag (H1029), anti-FLAG (F1804), anti-HA-tag (H3663) all from Sigma-Aldrich, anti-lgr5 (GPCR) (75732) from Abcam, HSP90 α/β (sc13119) from Santa Cruz Biotechnology, p-p38 MAPK α (Thr180, Tyr 182) (MA5-15177) from Invitrogen and anti-phospho-p38α (MAB8691) from R&D Systems. Rabbit IgG HRP and Mouse IgG HRP (#NA934V and #NA931V, GE Healthcare, respectively) were used as secondary antibodies and revealed using the ECL-plus chemiluminescence reagent (RPN2232, GE Healthcare). Densitometric evaluation was performed by ImageJ software.

### Co-immunoprecipitation

Co-IP was carried out as previously described^10^. Cells were lysed with the Nuclear Extraction Kit (ab113474, Abcam) according to the manufacturer’s instructions. Primary antibodies: p38α (#8690, Cell Signaling), β-catenin (#9562, Cell Signaling), and p-p38 MAPK α (Thr180, Tyr 182) (MA5-15177, Invitrogen). IgG was used as a negative control.

### Annexin V staining

2 × 10^4^ cells/plate were stained with Muse Annexin V and Dead Cell Reagent (Luminex MCH100105) according to the manufacturer’s instructions.

### Immunofluorescence

Cells were seeded on glass coverslips, fixed in 4% paraformaldehyde, and permeabilized using 0.01–0.1% Triton X-100. Coverslips were incubated with the indicated primary antibodies and then with Alexa Fluor 488 (#A-11094, Thermo Fisher Scientific) and 647 (#A-32728) secondary antibodies; nuclei were counterstained using DAPI (D9542, Sigma-Aldrich). Slides were sealed using Vectashield Mounting Medium (#H1000, Vector Laboratories). Images were acquired using a Zeiss fluorescence microscope. Primary antibodies: p38α (#8690, Cell Signaling) and β-catenin (#9562, Cell Signaling).

### Quantitative real-time PCR

RNA extraction and real-time PCR were performed as previously described^10^. Primer sequences are listed in Supplementary Table 1.

### Histology and immunohistochemistry

Tissue specimens were formalin-fixed in 4% buffered formalin, embedded in paraffin, sectioned at 4 mm thickness, and stained with hematoxylin and eosin. Additional sequential sections (3–5 μm) were cut and used for immunohistochemical analysis. Sections were dewaxed and rehydrated in dH_2_O. Endogenous peroxidase activity was blocked by incubation in 3% hydrogen peroxide for 10 min. Antigen retrieval was conducted in 10 mM sodium citrate buffer (pH 6.0) for 30 min. Sections were incubated overnight with the primary antibodies: p-p38α (1:100, M45-15177, Thermo Fisher Scientific), β-catenin (1:400, 9562, Cell Signaling), c-Myc (1:100, #9402, Cell Signaling), cyclin D1 (1:50, #2978, Cell Signaling), and CD44v6 (1:250, AB2080, Merck Millipore). Then, they were incubated with secondary biotinylated antibodies and subsequently with streptavidin–biotin–peroxidase (Envision + System HRP anti-rabbit and anti-mouse, K8002, Agilent). Samples were developed with DAB and mounted with permanent mounting media. Negative controls were used in each experiment. p-p38α, β-catenin, c-Myc, Cyclin D1, and CD44v6 immunoreactivity was evaluated by a semiquantitative approach by two independent pathologists, in a blinded manner, who scored the percentage of positive-stained cells and the intensity of the staining (0: absent, 1: mild and focal, 2: moderate, 3: intense and diffuse).

Immunocytochemical analysis was performed on cytospins using p-p38 MAPK α (Thr180, Tyr 182) (MA5-15177) (1:100) from Invitrogen. Single staining was revealed using a biotin–streptavidin system (Dako) and detected with 3-amino-9-ethylcarbazole (Dako). Nuclei were counterstained with aqueous hematoxylin (Sigma).

### Chromatin immunoprecipitation

Chromatin isolated from HT29 cells and CRC-SCs was subjected to chromatin immunoprecipitation using the MAGnify Chromatin Immunoprecipitation System (492024, Thermo Fisher Scientific) according to the manufacturer’s instructions. Chromatin was sonicated to a fragment length of about 200–500 bp and immunoprecipitated with 1 µg of rabbit IgG: p38α (#8690, Cell Signaling) and β-catenin (#9562, Cell Signaling). For re-ChIP, immune complexes were eluted with elution buffer (TE buffer, 10 mM DTT) for 30 min at 37 °C, diluted with the dilution buffer provided in the kit, and subjected to immunoprecipitation with a second antibody of interest. Primer sequences are listed in Supplementary Table 1.

### In vitro pull-down assay

His-β-catenin recombinant human protein was incubated with GST-p38α fusion protein. p300 (302-530)-GST fusion protein was used as a positive control. Fusion proteins were precipitated with Dynabeads His-Tag Isolation and Pulldown (10104D, Thermo Fisher Scientific) according to the manufacturer’s instructions. Primary antibodies: polyHistidine (H1029, Sigma-Aldrich) and GST (#2625, Cell Signaling). Rabbit IgG HRP and Mouse IgG HRP (#NA934V, #NA931V, GE Healthcare, respectively) were used as secondary antibodies and revealed using the ECL-plus chemiluminescence reagent (RPN2232, GE Healthcare).

### Cell sorting

For CRC-SC sorting, cells were collected, washed in PBS, and stained for 1 h at 4 °C with conjugated antibodies specific for CD44v6 (2F10 APC, mouse IgG1; R&D Systems) or a corresponding isotype-matched control (IMC). Dead cell exclusion was performed by using 7-AAD (0.25 µg/1 × 10^6^ cells, BD Biosciences). Cells were washed with 2% BSA and 2 mM EDTA in PBS and filtered with a 70 µm mesh to prevent cell clogging. Isolation of CD44v6^low^ and CD44v6^high^ cells was performed by using the FACSMelody cell sorter. Post-sorting analysis was performed to verify the purity of sorted populations.

### Karyotyping protocol

CRC-SCs were seeded at high density (2 × 10^6^ cells/ml). After 24 h, colcemid-treated CRC-SCs were incubated in a hypotonic solution, fixed in a chilled fixative solution, and then washed extensively. Chromosomes were counted using an Olympus microscope.

### Mutation analysis

For targeted deep DNA re-sequencing, the sequencing library was prepared as previously described^25^. MSI analysis was performed with a reference panel of five fluorescent dye-labeled microsatellite primers (NR-21, BAT-25, MONO-27, NR-24, BAT-26) using the MSI Analysis System kit (MD1641, Promega). Amplified fragments were detected by loading the PCR products for capillary electrophoresis using an ABI Prism 3500 Genetic Analyser and the POP-4 polymer (4393710, Applied Biosystems) according to the manufacturer’s instructions. MSI status was determined upon analysis with GeneMapper software, Version_4.1 (Applied-Biosystems).

### Cellular assays

For cell viability assays, viability was assessed using the CellTiter-Glo Luminescent Cell Viability Assay Kit (G7570, Promega) according to the manufacturer’s instructions. The luminescent signal was read using a SPECTROstar Omega microplate reader (BMG Labtech). The CellTiter 96^®^ AQueous One Solution Cell Proliferation Assay (G3580, Promega) (MTS) was performed according to the manufacturer’s instructions and analyzed by using the GDV MPT reader (DV 990 BV6).

For cell death assays, cell death was assessed by cell counting as previously described^17^. Human intestinal 3D cultures and APC^Min/+^ mouse intestinal organoids were stained using the LIVE/DEAD^®^ Cell Imaging Kit (R37601, Thermo Fisher Scientific) according to the manufacturer’s instructions.

For clonogenic assays, dissociated CRC-SCs were plated in triplicate at 500 cells/well suspended in 0.3% agarose over a layer of 0.5% agarose and treated as indicated.

For motility assays, 1 × 10^4^ control or treated CRC-SCs were suspended in 200 μl of non-supplemented stem cell medium and plated into the upper wells of Matrigel-coated Boyden chambers containing 8 μm diameter polycarbonate membranes (CLS3422-48EA, Corning). Lower wells contained 600 μl of stem cell medium supplemented with 20 ng/ml EGF and 10 ng/ml basic FGF and the relevant drugs.

For proliferation assays, CRC-SCs treated as indicated were analyzed to determine the percentage of proliferating cells based on Ki67 expression using the Muse Ki67 Proliferation Kit (MCH100114, Merck Millipore) according to the manufacturer’s instructions.

For the luciferase assay, HEK293 cells were lysed with 100 µl Passive Lysis (E1910, Promega) and the assay was performed according to the manufacturer’s instructions.

Analysis of p38α kinase activity was performed using the ADP-Glo Kinase Assay (V6930, Promega) according to the manufacturer’s instructions. p38α active protein (25 ng, V2701, Promega) was assayed in a kinase reaction buffer with 50 µM ATP and varying concentrations of human recombinant β-catenin (0.5, 1, and 1.5 μg). 1 μg of p38 peptide substrate was used as a control. The generated luminescence was measured using a luminometer (SPECTROstar Omega microplate reader, BMG Labtech).

### ELDA

CD44v6^low^- and CD44v6^high^-enriched cells were plated at 1, 2, 4, 8, 16, 32, 64, and 128 cells per well in 96-well plates. Clonal frequency was calculated using the extreme limiting dilution analysis (ELDA) tool (http://bioinf.wehi.edu.au/software/elda/index.html).

### In vivo studies

For in vivo studies, normal, adenoma, and adenocarcinoma colon mucosa tissues were obtained from C57BL/6 mice (*n* = 12), APC^Min/+^ mice (*n* = 12), and APC^Min/+^ mice (*n* = 24) treated with 14 mg/kg of AOM (A5486, Sigma-Aldrich), respectively. Four-month-old APC^Min/+^ male mice were administered with AOM (14 mg/kg body weight) once a week for 5 weeks; one month later, they were subjected to daily intraperitoneal injections of the p38α inhibitor SB202190 (0.05 μmol/kg body weight) or DMSO for 14 days and then sacrificed. Body weight was recorded daily. Procedures involving animals and their care were conducted in conformity with the institutional guidelines that comply with national and international laws and policies.

### TCGA PanCancerAtlas data source and meta-analysis

To study the association between p38α mRNA expression levels and CRC aggressiveness, RNA-seq gene expression data (*Z*-scores) of 592 CRC patients and TNM stage clinical data of 580/592 patients were obtained from TCGA PanCancerAtlas through the cBioPortal website^50^. Patients were stratified based on p38α mRNA *Z*-score into two groups with high (>median, *n* = 296/592) or low (≤median, *n* = 296/592) p38α mRNA expression. Statistical analysis was performed using R (version 3.6.2), an open-source freely available software environment for statistical computing and graphics. Survival curves of stage III CRC patients (*n* = 171) were assessed according to the Kaplan–Meier method, and DSF and PFS were used as the endpoint. Differences between stage III CRC patients with high p38α mRNA (*n* = 80/171) and low p38α mRNA (*n* = 91/171) were assessed using the log-rank test and R packages “*survival*” and “*survminer*”^51–53^.

### In silico prediction analysis

In silico prediction analysis was performed using DISPHOS 1.3, NETPHOS 3.1, Phosida, iPTMne, and Phosphosite Plus servers.

### Quantification and statistical analysis

The statistical significance of the results was analyzed using the Student’s *t*-tail test, and *P* < 0.05 was considered statistically significant. Results are representative of at least three independent experiments.

## Supplementary information

supplementary table

supplementary figure legends

Supplementary Figure 1

Supplementary Figure 2

Supplementary Figure 3

Supplementary Figure 4

Supplementary Figure 5

Supplementary Figure 6

Supplementary Figure 7

## Data Availability

All data are available upon request to the corresponding author.
