# Supplementary material for: Pharmacological targeting of the novel β-catenin chromatin-associated kinase p38α in colorectal cancer stem cell tumorspheres and organoids
Source: Cell Death Dis. 2021 Mar 25;12(4):316. doi: 10.1038/s41419-021-03572-4 (PMC7994846; doi:10.1038/s41419-021-03572-4)
Supplement: Supplementary file 2 — supplementary figure legends [file 41419_2021_3572_MOESM2_ESM.pdf]

Supplementary figure legends

Supplementary Figure 1. Characterization of patient-derived stage III CRC-SCs

(A) Mutation, chromosomal (CIN) and microsatellite instability (MSI) analysis of patient-derived stage III CRC-SCs (#40, #8, #21, #9) F=female; M=male; R=rectum; S=sigmoid.

(B) Immunoblot analysis showing the expression of several stem cell markers in CRC-SCs #21, HCEC-1CT normal colonocytes, HCT116 and HT29 CRC cell lines. β-actin was used as a loading control.

(C) Immunoblot analysis showing p-p38α levels in CRC-SCs. HSP90 was used as a loading control.

Supplementary Figure 2. p38α and β-catenin subcellular co-localization

(A) Left panels: Immunoblot analysis showing p38α and β-catenin subcellular localization in HCEC-1CT and HT29 cells under different culture conditions. Cells were serum-starved for 48 h and then switched to a serum-containing medium with or without LiCl (10 mM) for 4 h. Right panels: Densitometric analysis of the indicated proteins normalized against the loading control.

(B) Left panels: Immunofluorescence analysis showing p38α and β-catenin subcellular localization in HCEC-1CT and HT29 cells under serum starvation and after serum addition. Right panel: Quantification of p38α and β-catenin co-staining.

(C) Upper panels: Immunoblot analysis showing p38α and β-catenin subcellular localization in CRC-SCs #40, #9, and #21 treated with PRI-724 (1 µM) for 24 h or with Wnt3a (50 ng/ml) and/or LiCl (10 mM) for 4 h. Lower panels: Densitometric analysis of the indicated proteins normalized against the loading control.

(A, C) Lamin B1: nuclear loading control; PDI: cytoplasmic loading control. UT=untreated; N=nucleus; C=cytoplasm; LiCl=lithium chloride.

Statistical analysis was performed using Student's t-tail test; *P<0.05 vs UT; $^{\#}$P<0.05 vs. serum-starved cells was considered statistically significant.

Supplementary Figure 3. Functional interaction between p38α and β-catenin in patient-derived stage III CRC-SCs and mouse models

(A) Co-immunoprecipitation of endogenous p38α and β-catenin in patient-derived stage III CRC-SCs #40.

(B) Co-immunoprecipitation of endogenous p38α and β-catenin (β-cat) in nuclear and cytoplasmic fractions of patient-derived stage III CRC-SCs #40. Lamin B1: nuclear loading control; PDI: cytoplasmic loading control.

(C) Immunoblot analysis of the indicated proteins in C57BL/6 mice and in $APC^{Min/+}$ mice treated or not with azoxymethane.

(D) Densitometric analysis of co-immunoprecipitated p38α and β-catenin in nuclear and cytoplasmic fraction from colon tissues isolated from 12 C57BL/6 mice and 12 AOM-treated $APC^{Min/+}$ mice.

(E) ChIP with anti-p38α and anti-β-catenin antibodies in CRC-SCs #21 and #8. Quantification was done using the % input method.

N=nucleus; C=cytoplasm, AOM=azoxymethane. Statistical analysis was performed using Student's t-tail test; *P<0.05 was considered statistically significant.

Supplementary Figure 4. Prediction analysis of phosphorylation sites on β-catenin amino acid sequence

Schematic representation of the human β-catenin protein highlighting functional domains and binding sites of the main interactors. *In silico* prediction analysis was performed using five different servers: DISPHOS 1.3, NETPHOS 3.1, Phosida, iPTMnet, and Phosphosite Plus.

Supplementary Figure 5. Involvement of p38α active form in p38α-β-catenin complexes

(A) Immunoprecipitation of whole-cell lysates with an antiserum against p38α followed by immunoblotting with anti-β-catenin and anti-p-p38 antibodies in CRC-SCs #21.

(B) Immunoprecipitation of whole-cell lysates with an antiserum against β-catenin followed by immunoblotting with anti-p38α and anti-p-p38 antibodies in CRC-SCs #21.

(C) Immunoprecipitation of whole-cell lysates with an antiserum against p-p38α followed by immunoblotting with anti-β-catenin and anti-p38α antibodies in CRC-SCs #9.

(A-C) Input corresponds to 10% of the lysate.

Supplementary Figure 6. CRC-SC sorting of CD44v6^{low}- and CD44v6^{high}-enriched populations

Flow cytometry gating strategy (single cells/viable cells gates) and cell sorting of top 20% CD44v6^{low} versus CD44v6^{high} CRC-SCs #8, #9, #21, and #40. Insets indicate the specific isotype-matched control (IMC).

Supplementary Figure 7. p38α inhibition sensitizes patients-derived stage III CRC-SCs to chemotherapeutics

(A) Quantification of cell viability by Cell Titer Glo in CRC-SCs #40, #8, and #9 pre-treated with ralimetinib (10 μM) for 48 h and then treated with 5-FU (2 μM), CDDP (30 μM), CPT-11 (30 μM), or trametinib (1 nM) for another 24 h in the presence of ralimetinib.

(B) Quantification of cell death by trypan blue staining in CRC-SCs #40, #8, and #9 treated as described in (A).

(C) Colony-forming ability of CRC-SCs #40, #8, #9 seeded onto double-layer soft agar and treated as described in (A). Data represent the percentage of colonies relative to DMSO-treated cells.

(D) Migratory ability of growth factor-starved CRC-SCs #40, #8, and #9 placed in the inner chamber of transwell plates and treated with the indicated compounds for 16-20 h. Migrating cells were fixed and counted under a fluorescence microscope.

(E) Graph summarizing the percentage of Ki67-positive cells detected by flow cytometry analysis in CRC-SCs #40, #8, and #9 treated as described in (A).

(F) Graph summarizing the percentage of apoptotic cells (early + late) detected by flow cytometry analysis of annexin V staining in CRC-SCs #40, #8, and #9 treated as described in (A).

Blue dots: #40; red dots: #9; green dots: #8. 5-FU=5-fluorouracil; CDDP=cisplatin; CPT-11=irinotecan; Tram=trametinib. Statistical analysis was performed using Student’s t-tail test; *$P<0.05$: treatments vs. control (DMSO); and $^{\#}P<0.05$: combined treatment vs. corresponding single treatments.
