# Supplementary figures and images for: Pharmacological targeting of the novel β-catenin chromatin-associated kinase p38α in colorectal cancer stem cell tumorspheres and organoids
Source: Cell Death Dis. 2021 Mar 25;12(4):316. doi: 10.1038/s41419-021-03572-4 (PMC7994846; doi:10.1038/s41419-021-03572-4)

Figure S1

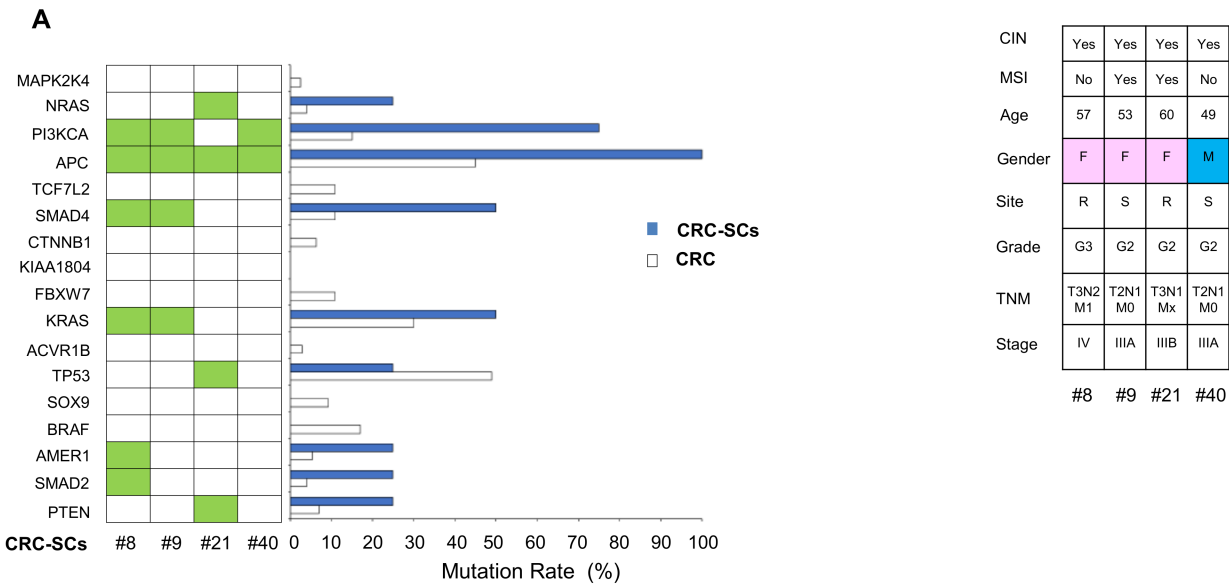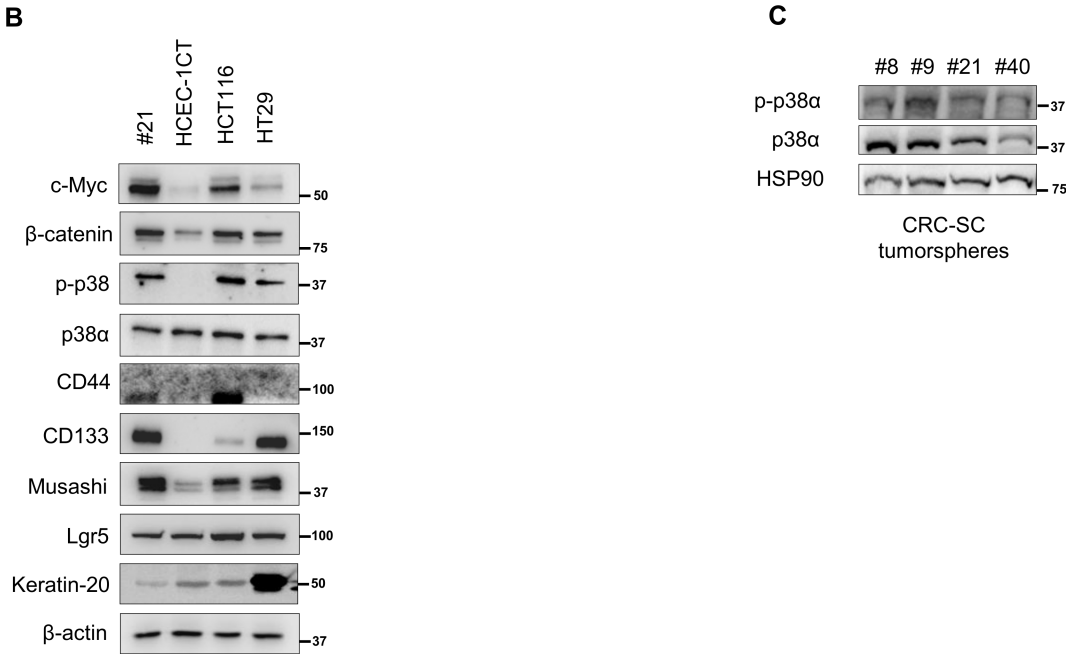

Supplement: Supplementary file 3 — Supplementary Figure 1 [file 41419_2021_3572_MOESM3_ESM.pdf]

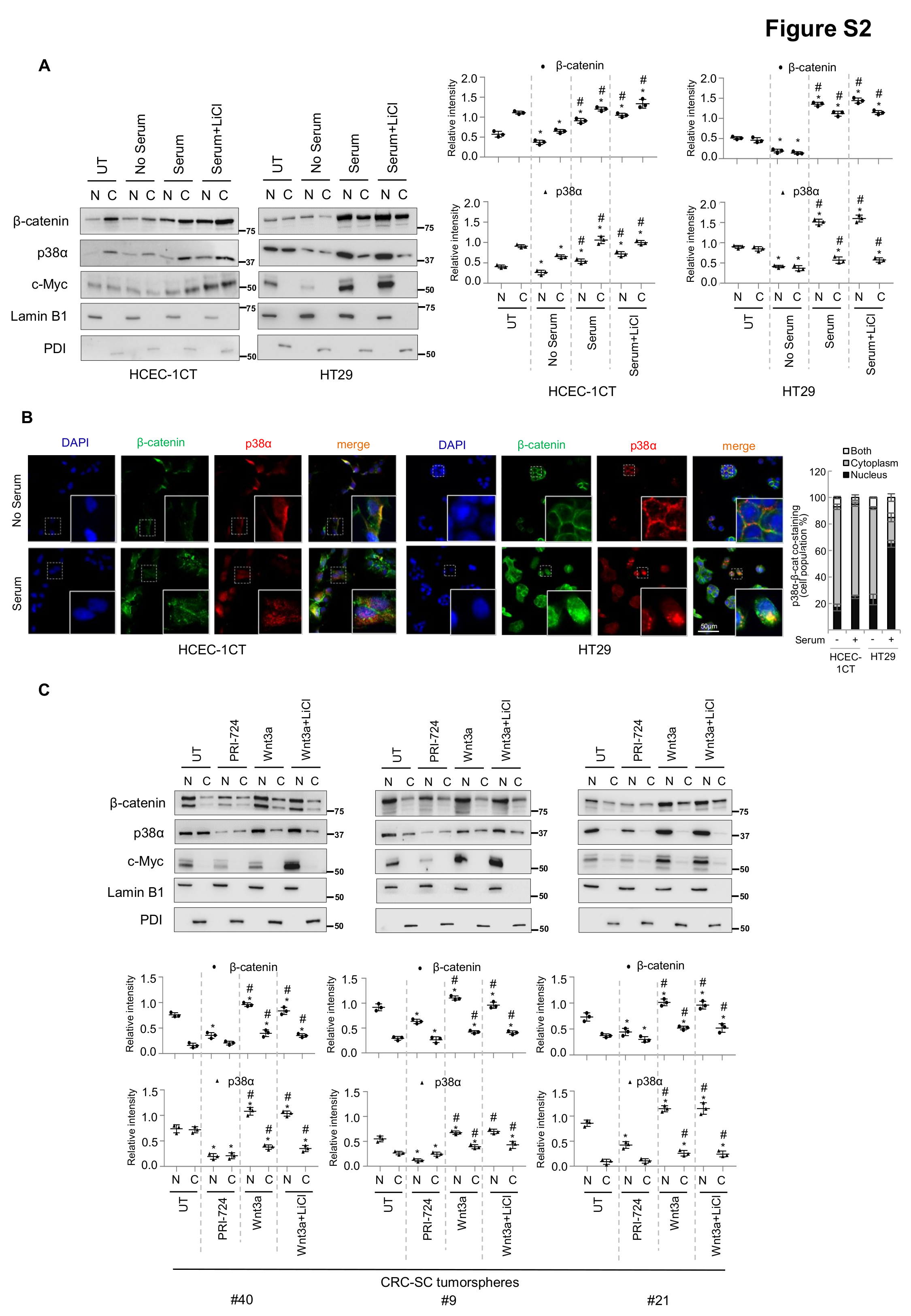

Supplement: Supplementary file 4 — Supplementary Figure 2 [file 41419_2021_3572_MOESM4_ESM.tif]

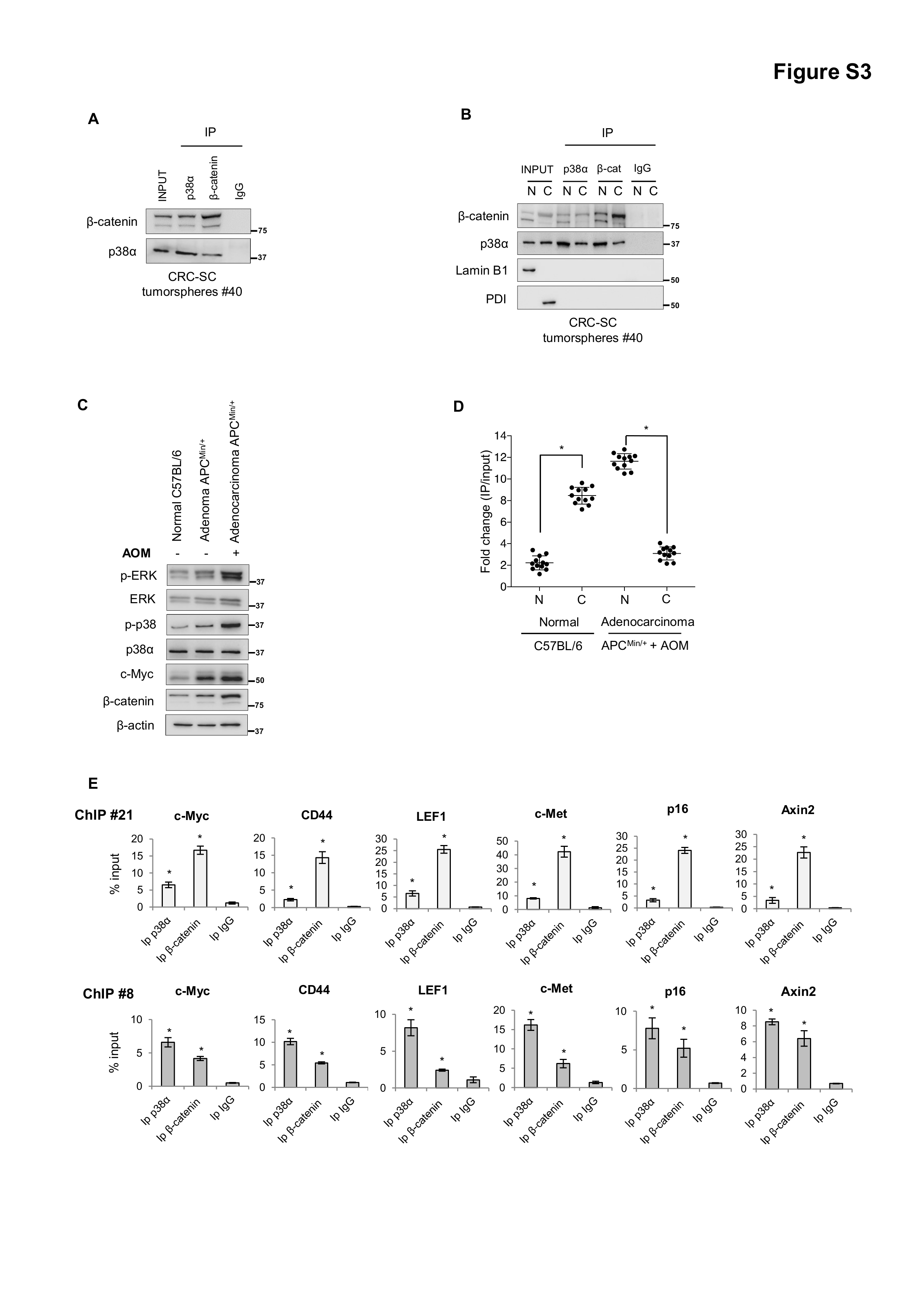

Supplement: Supplementary file 5 — Supplementary Figure 3 [file 41419_2021_3572_MOESM5_ESM.tif]

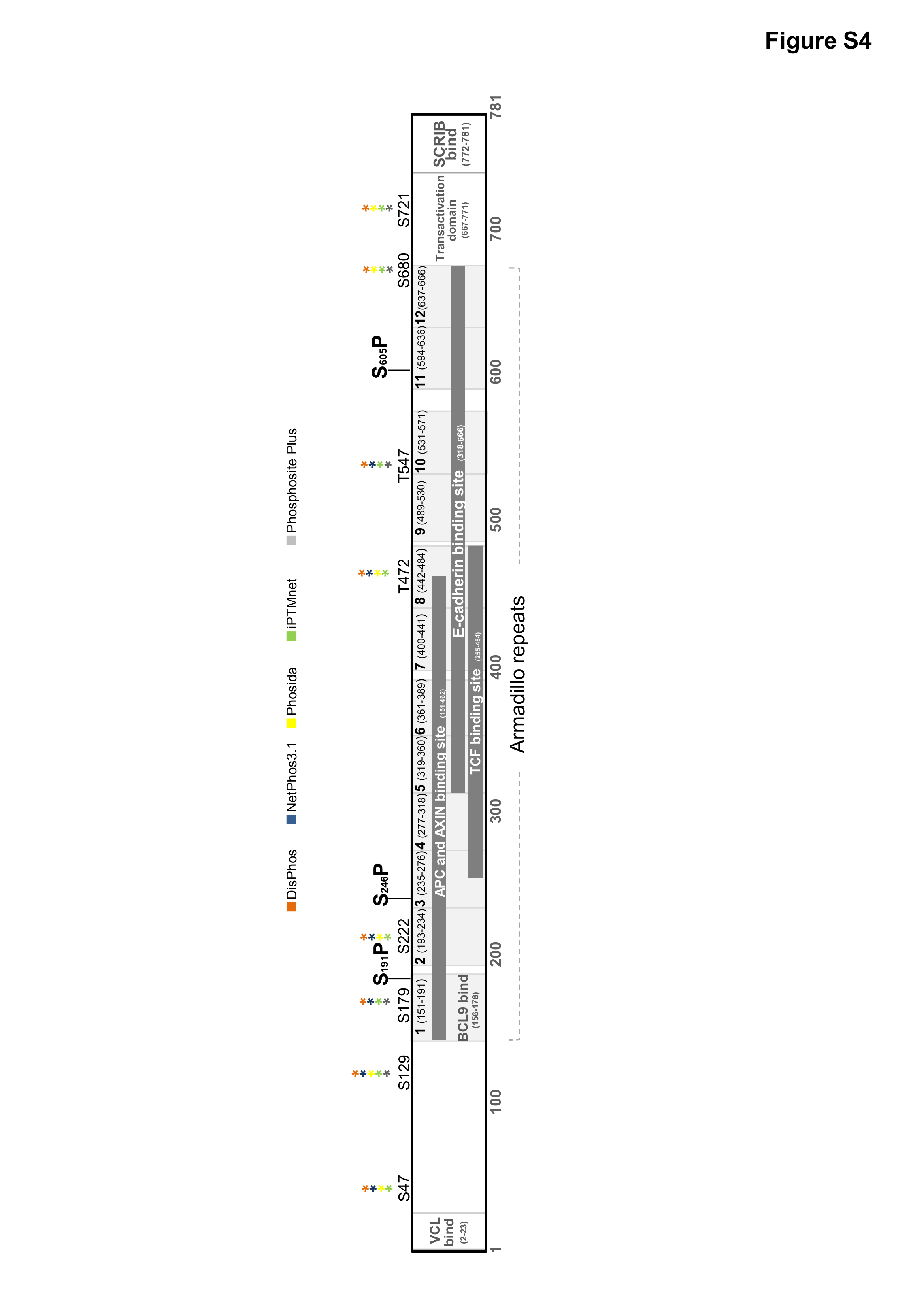

Supplement: Supplementary file 6 — Supplementary Figure 4 [file 41419_2021_3572_MOESM6_ESM.tif]

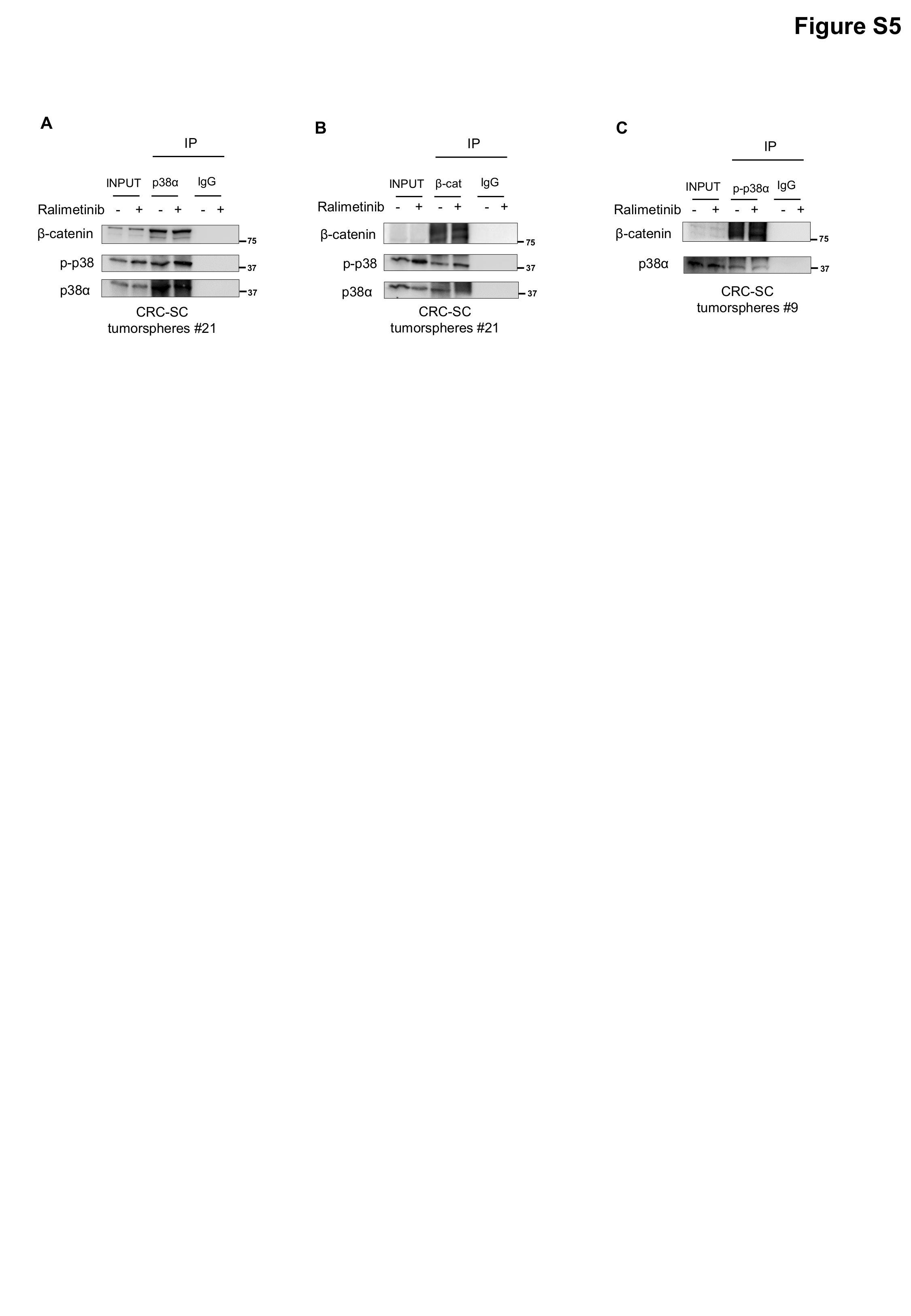

Supplement: Supplementary file 7 — Supplementary Figure 5 [file 41419_2021_3572_MOESM7_ESM.tif]

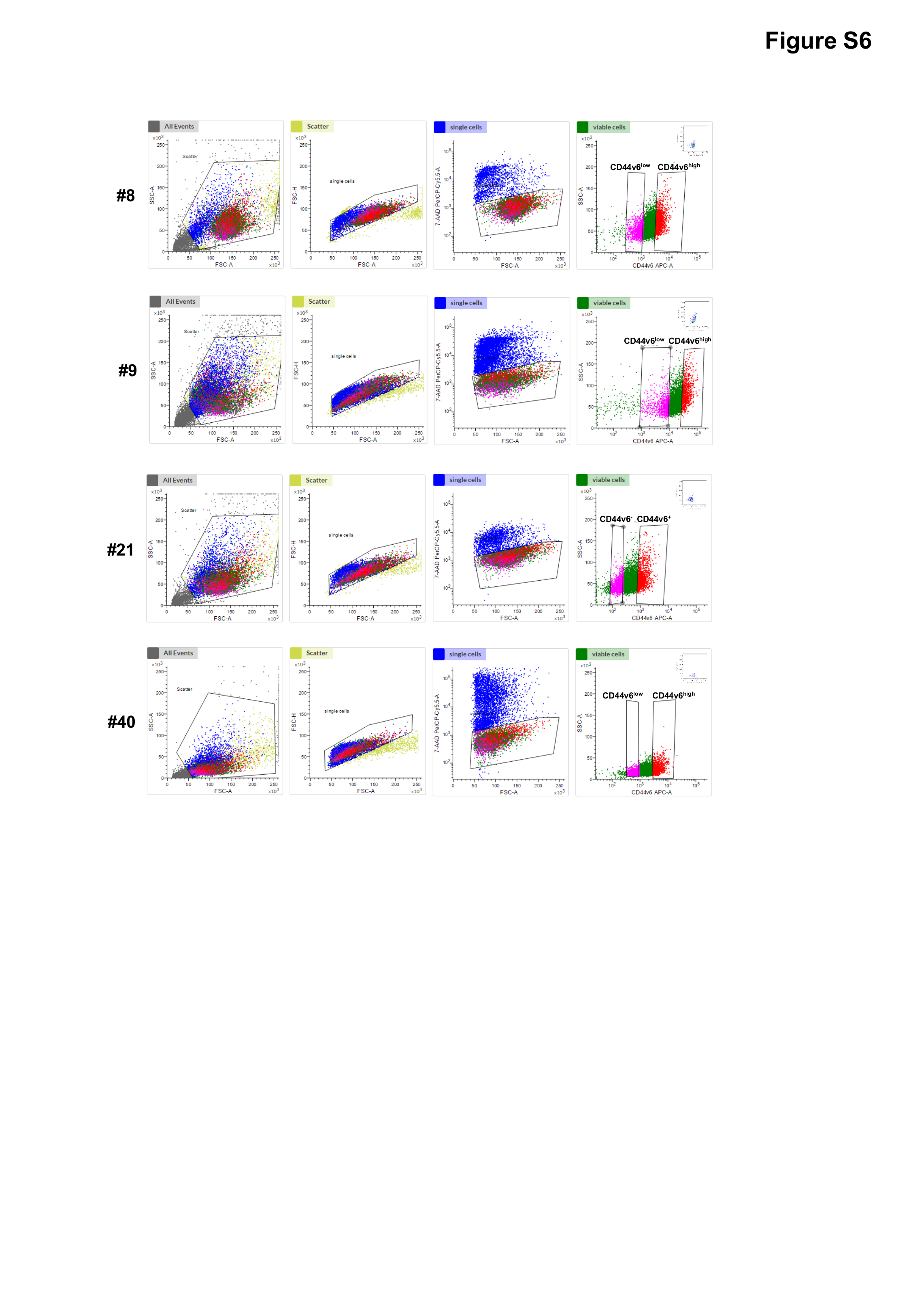

Supplement: Supplementary file 8 — Supplementary Figure 6 [file 41419_2021_3572_MOESM8_ESM.tif]

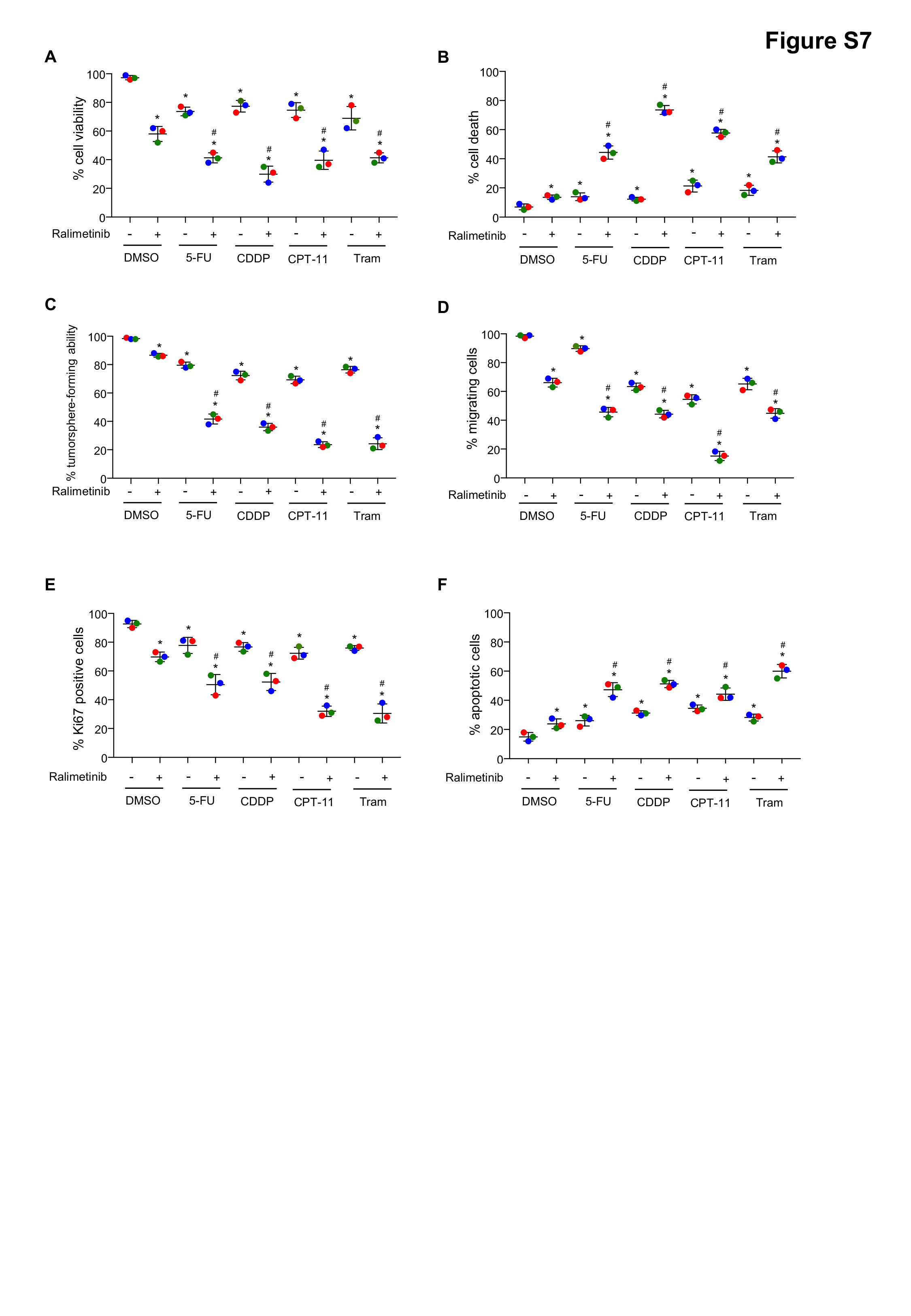

Supplement: Supplementary file 9 — Supplementary Figure 7 [file 41419_2021_3572_MOESM9_ESM.tif]
